# Differentiating *Staphylococcus aureus* from *Escherichia coli* mastitis: *S*. *aureus* triggers unbalanced immune-dampening and host cell invasion immediately after udder infection

**DOI:** 10.1038/s41598-017-05107-4

**Published:** 2017-07-06

**Authors:** Juliane Günther, Wolfram Petzl, Isabel Bauer, Siriluck Ponsuksili, Holm Zerbe, Hans-Joachim Schuberth, Ronald M. Brunner, Hans-Martin Seyfert

**Affiliations:** 1 Institute for Genome Biology, Leibniz Institute for Farm Animal Biology, 18196 Dummerstorf, Germany; 2 Clinic for Ruminants with Ambulance and Herd Health Services, Centre for Clinical Veterinary Medicine, Ludwig-Maximilians-University Munich, 85764 Oberschleißheim, Germany; 3 Immunology Unit, University of Veterinary Medicine Foundation, 30173 Hannover, Germany; 4 Gebr. Ewald GmbH, 98553 Nahetal-Waldau, Germany

## Abstract

The etiology determines quality and extent of the immune response after udder infection (mastitis). Infections with Gram negative bacteria (e.g. *Escherichia coli*) will quickly elicit strong inflammation of the udder, fully activate its immune defence *via* pathogen receptor driven activation of IκB/NF-κB signaling. This often eradicates the pathogen. In contrast, Gram-positive bacteria (e.g. *Staphylococcus aureus*) will slowly elicit a much weaker inflammation and immune response, frequently resulting in chronic infections. However, it was unclear which immune regulatory pathways are specifically triggered by *S*. *aureus* causing this partial immune subversion. We therefore compared in first lactating cows the earliest (1–3 h) udder responses against infection with mastitis causing pathogens of either species. Global transcriptome profiling, bioinformatics analysis and experimental validation of key aspects revealed as *S*. *aureus* infection specific features the (i) failure to activating IκB/NF-κB signaling; (ii) activation of the wnt/β-catenin cascade resulting in active suppression of NF-κB signaling and (iii) rearrangement of the actin-cytoskeleton through modulating Rho GTPase regulated pathways. This facilitates invasion of pathogens into host cells. Hence, *S*. *aureus* mastitis is characterized by eliciting unbalanced immune suppression rather than inflammation and invasion of *S*. *aureus* into the epithelial cells of the host causing sustained infection.

## Introduction

Infection of the udder (mastitis) occurs frequently and is the most costly infection-related disease in dairy farming^1^. The outcome largely depends on the etiology^2^. Gram-negative pathogens, such as *Escherichia coli* (*E*. *coli*) provoke strong inflammation through a vigorous stimulation of cytokine synthesis, resulting in full activation of the local and generalized immune response of the host^3, 4^. This leads very often to eradication of the pathogen, albeit that some *E*. *coli* strains have been identified which may persist for some time in the udder^5^. *Staphylococcus aureus* (*S*. *aureus*) and other Gram-positive pathogens elicit a much weaker immune reaction of the udder and generally no strong systemic immune reaction (see reviews)^6, 7^. As a result these infections may very often become persistent^8^.

In recent years key aspects of the molecular mechanisms underpinning these fundamental differences in the pathogen-specific physiology of mastitis have been identified *in vitro* based on cell models. It was shown that the pathogen-specific immune defense reaction of the mammary epithelial cell (MEC)^9^ determines the ability of the udder to fighting off bacteria^10, 11^. Using MEC it was found *in vitro* that challenges with *E*. *coli*, but not *S*. *aureus* trigger a vigorous cytokine storm in these cells^12–16^. This key difference is due to the failure of *S*. *aureus* to activating signaling from the toll-like-receptors (TLR) in the MEC^11, 13, 17^. As a consequence, challenging these cells with *S*. *aureus* will not substantially activate the NF-κB complex of transcription factors, those well-known master regulators of immune gene expression^18^.

Despite this large body of evidence regarding the pathogen-specific differentiation of the immune response of the MEC *in vitro*, the determinants are still elusive distinguish *in vivo* the very early immune response of the udder in a pathogen-specific fashion. However, those *in vitro* studies suggest that already the first pathogen contacts during the first hour of infection differentiate the immune response of those epithelial cells in a pathogen-specific fashion^17^.

We tried previously to elucidate *in vivo* pathogen-specific differences in the immune response and compared transcriptome alterations in udder samples collected in a randomized trial in which mid-lactating heifers had been infected with either *E*. *coli*_1303_ or *S*. *aureus*_1027_ pathogens. However, only as late as 24 h post infection (pi) did the *E*. *coli* infection result in statistically significant alterations of the expression of selected candidate genes in the milk-producing parenchyma, while the *S*. *aureus* infection had to last for 72 h or longer to yielding significant regulation of some candidate genes^19^. Global transcriptome profiling of *S*. *aureus* infected udder samples identified only 5 regulated gene loci as early as 12 h pi, but not any more from later time points. For comparison, *E*. *coli* infection had regulated the expression of 1048 loci, all at 24 h pi^4, 20^. These longer term mastitis models had in addition the problem, that the udder response against *E*. *coli* infection was confounded by strong systemic responses^4, 20^. Others profiled in cattle or goat the response of the udder after infecting with *S*. *aureus* only^21–23^. While these data show some upregulation of cyto- and chemokine encoding genes, the extent of their regulation cannot be evaluated against the full immune responsive capacity of the animals, since the direct comparison against an infection with *E*. *coli* under identical experimental settings had not been provided.

We therefore developed an alternative mastitis model by sequentially infecting udder quarters of healthy mid-lactating heifers with the same high dose (10^6^ live bacteria/udder quarter) of defined *E*. *coli*_*1303*_ or *S*. *aureus*_*1027*_ pathogens and sampled the quarters at 1, 2 and 3 h pi. We described the model and its clinical aspects in a companion paper^24^ and indicated that neither infection was accompanied by any signs of a systemic reaction (absence of fever, no alteration of blood leucocyte counts) or udder swelling and changes in the counts of somatic milk cells. We also validated, based on a limited set of inflammation-related candidate genes that infections with both pathogens had provoked significant modulations of immune gene expression and that expectedly *E*. *coli* had provoked a stronger inflammatory response than *S*. *aureus*.

We have now exploited those samples for global transcriptome profiling to get a naïve and unbiased view on the very early immune response of the udder against invading *E*. *coli* and *S*. *aureus* strains. We found that *S*. *aureus*, but not *E*. *coli* infection quickly triggers prevailing immune evasive mechanisms in the udder, through distinct immunosuppression and invasion of the pathogen into the epithelial cells of the host.

## Results

### Validation of microarray data

We have exploited for this study tissue samples from the gland cistern (GC) which had been collected in a previous infection trial and from which the expression of a set of candidate genes had already been determined with RT-qPCR^24^. Hence, we compared for 8 genes (MX2, IL10, S100A9, TNF, IL8, IL6, CCL20, LCN2) the previously measured data with the extent of mRNA modulation as measured in the current analysis using microarray hybridization. We found a highly significant positive correlation (r, 0.74; P < 0.0001) between both methods based on 192 individual measurements conducted with each of both methods (Supplementary Figure 1).

### *E*. *coli* regulated faster and more genes than *S*. *aureus*

**Figure 1:**
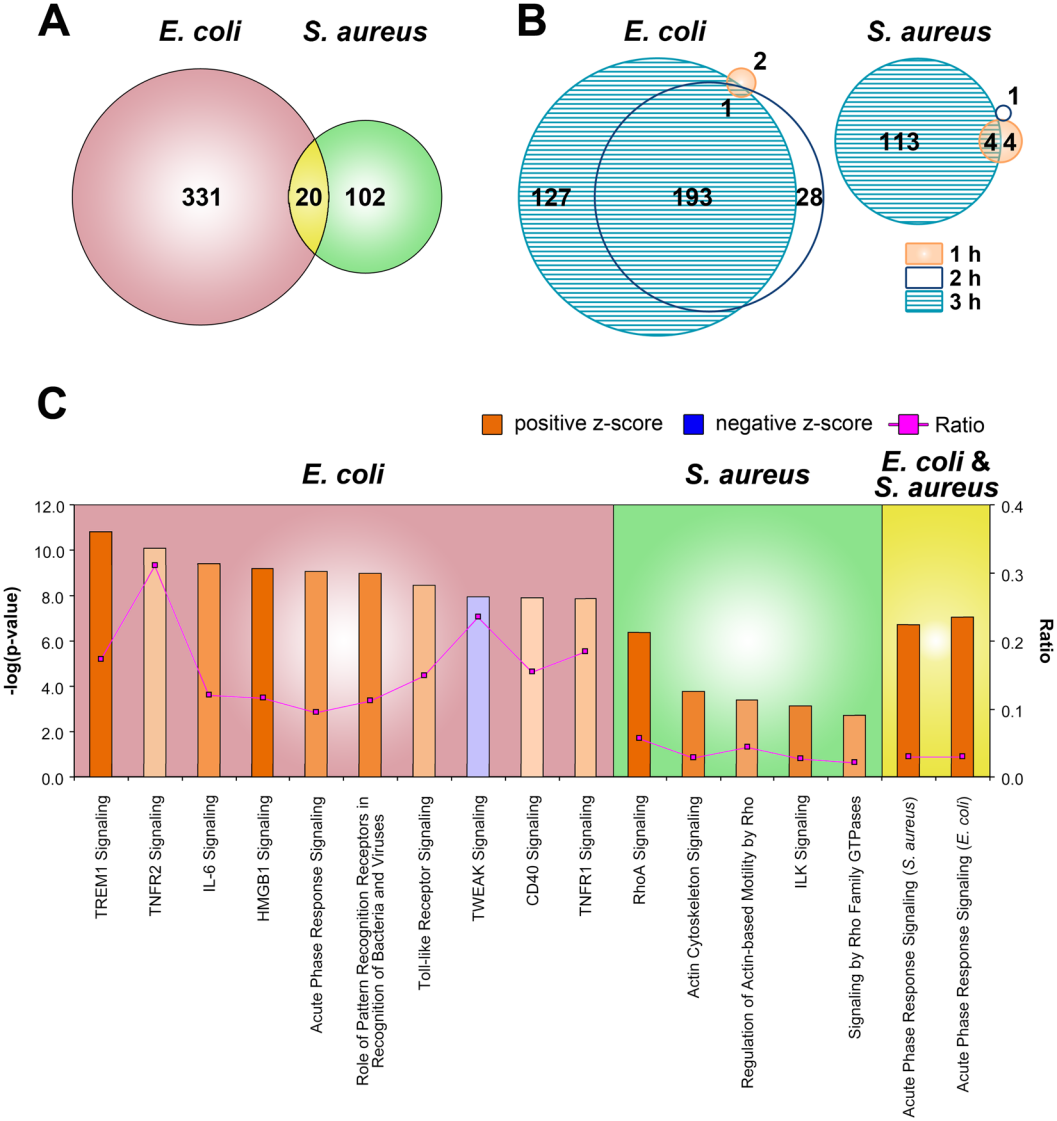
Number and time course of differentially activated genes during *E*. *coli* and *S*. *aureus* mastitis and their contribution to regulatory pathways. Venn diagrams of (**A**) total number of loci regulated either by *E*. *coli* or *S*. *aureus* infection and (**B**) number of loci regulated at 1, 2 or 3 h pi during either *E*. *coli* or *S*. *aureus* infection. (**C**) Ingenuity based identification of key signaling pathways as regulated by either *E*.*coli* or *S*. *aureus* infection or by both pathogens. The ordinate indicates the logarithm of the statistical significance (p-value). The z-score represents the likelihood that the pathway is regulated (red, up-; blue, down-regulated), with more intense colors reflecting higher significance. Ratio indicates the proportion of DEGs found in the current data set relative to the total number of genes contributing to that pathway.

*E*. *coli* infection modulated the mRNA abundance of 351 locus specific transcripts during the first 3 h of udder infection. The Ingenuity program related 291 of them to known genes subsequently referred to as differentially expressed genes (DEGs). In contrast, *S*. *aureus* infection changed the expression of only 122 loci 98 of which could be associated with known genes (Fig. 1A). Only 20 loci including 16 DEGs were regulated by infection with both pathogen species (Table 1). Supplementary Table 1 lists all genes regulated exclusively by either *E*. *coli* or *S*. *aureus* infection. Sorting the regulated genes according to the family of factors which they encode shows that *E*. *coli* regulated above average cytokines, nuclear receptors, micro RNAs and transmembrane receptors (Table 2). The regulated genes included 30 transcription factors and regulators. They included NF-κB factors and their inhibitors, C/EBP factors and NFIL3. The latter factor has previously been highlighted as a major regulator during early *E*. *coli* induced mastitis^20^. Neither of them was regulated during the *S*. *aureus* infection albeit that this challenge also regulated the expression of 8 transcription regulators. Among these, only the factors JUNB and EGR1 were also regulated during the *E*. *coli* infection (Table 1). The comparison of the extent of changes in the mRNA abundance derived from those 16 DEGs having been regulated by both types of infection shows that *E*. *coli* induced 8 of them more strongly, while the others were induced to approximately the same extent (Table 1).

**Table 1:** Genes induced by *E*. *coli* and *S*. *aureus* infection.

Symbol	Gene	Induction [fold]		Factor function	Upstream regulator		
	Name	*E*. *coli*	*S*. *aureus*				
IL1RN	interleukin 1 receptor antagonist	10.3	1.7	cytokine/antagonist	PGN	IFNG	P38
TNF	tumor necrosis factor	8.3	1.9	cytokine	PGN	IFNG	P38
CCL8	chemokine (C-C motif) ligand 8	6.3	2.4	cytokine	PGN	IFNG	P38
CXCL10	chemokine (C-X-C motif) ligand 10	2.0	1.6	cytokine	PGN	IFNG	P38
IL18	interleukin 18	1.6	1.6	cytokine	PGN	IFNG	
JUNB	jun B proto-oncogene	4.0	1.7	Transcription regulator	PGN	IFNG	P38
EGR1	early growth response 1	1.9	1.5	Transcription regulator	PGN	IFNG	P38
CD200	CD200 molecule	1.9	1.5	Immune inhibitor	PGN	IFNG	
CFB	complement factor B	5.3	1.8	peptidase		IFNG	
HP	haptoglobin	5.3	1.5	Membrane protection			P38
ALDH1A3	aldehyde dehydrogenase 1 family member A3	3.3	1.7	enzyme		IFNG	
MX2	MX dynamin-like GTPase 2	1.8	1.6	enzyme		IFNG	
AGMO	alkylglycerol monooxygenase	−1.6	−1.5	enzyme			
TRPC4	transient receptor potential cation channel, subfamily C, member 4	1.6	1.5	ion channel		IFNG	
SLC46A2	solute carrier family 46 member 2	3.3	1.6	transporter			
GJA1	gap junction protein alpha 1	1.6	1.7	transporter		IFNG	P38

PNG, peptidoglycan; INFG, interferon γ; P38, p38 mitogen-activated protein kinases.

**Table 2:** Pathogen specific modulation of factor families.

Factor family	*E. coli*	*S. aureus*
cytokine	15	1
enzyme	46	13
G protein coupled receptor	18	2
Growth factor	6	2
Ion channel	3	0
Kinase	6	6
Nuclear receptor	5	0
Micro RNA	13	1
Peptidase	10	1
Phosphatase	4	1
Transcription regulators	30	8
Transmembrane receptor	21	1
Transporter	6	3

Columns indicate the numbers of DEGs contributing to the respective factor family, as identified in *E*. *coli* or *S*. *aureus* infected specimen.

Considering the time course of immune activation, we found that *E*. *coli* modulated the mRNA abundance from only 3 loci during the first h of infection (Supplementary Table 1) including the upregulated expression IL1RN (interleukin 1 receptor antagonist) known to antagonize IL1 signaling^25^. *S*. *aureus*, however very quickly modulated the mRNA abundance stemming from 8 loci already during the first h of infection (Supplementary Table 1). The upregulated DEGs included two chemokine ligands (CXCL10, CCL8) relevant for immune cell recruitment, but also NPR3 (natriuretic peptide receptor C). The ligand for this receptor is an antagonist of TNF-function and IL6 secretion^26^. Immune dampening is also indicated by the downregulated expression of TAGAP (T-cell activation GTPase-activating protein), a known activator of T-cell function^27^.

*E*. *coli* infection regulated the majority of all DEGs already at 2 h pi (Fig. 1B; 222 loci from among 351 = 63%; Supplementary Table 2). This compares to only 5 DEGs regulated by the *S*. *aureus* infection that shortly after infection (*e*.*g*. 5/122 = 4.1%). Most of these early regulated genes were also upregulated at 3 h pi. MX2 (MX dynamin-like GTPase 2) was the single DEG having been upregulated (1.6 fold) during the 2^nd^ h of the *S*. *aureus* infection. For comparison, *E*. *coli* infection up-regulated the expression of MX2 by 1.8 fold during the 3^rd^ h of infection.

### *E*. *coli* infection dominantly regulated pathways eliciting inflammation while *S*. *aureus* triggered cytoskeletal rearrangements

The top Canonical Pathways having solely been addressed by the *E*. *coli* infection regulated genes were all related to inflammation and pathogen related signaling (Fig. 1C). Highest significance-probabilities were attributed to the pathways ‘signaling through TREM1’ (triggering receptor expressed on myeloid cells), TNFR2 (tumor necrosis factor receptor subfamily, member 1B) and IL6. They also included ‘signaling mediated through pattern recognition receptors’, such as TLR2 (toll-like receptor 2) and other receptors, such as CD40 and ICAM1 (intercellular adhesion molecule 1).

Acute Phase Signaling emerged as the only from the multitude of annotated pathways having been activated by those 16 genes found to be regulated during the infection with both pathogens. Six of them (*e*.*g*. 37.5%) contribute to this pathway. The respective DEGs included in the *E*. *coli* infected samples SOCS1, SOCS3, IL1A, NFKBIE, CP, SERPINA3, IL6, NFKB2, NFKB1, FOS, NFKBIA, SOD2, IL1B, LBP, SERPINE1, and IL1RAP. In contrast, relevant DEGs found in those *S*. *aureus* infected tissues were IL18, HP, IL1RN, CFB, and TNF.

The DEGs regulated solely by the *S*. *aureus* infection can almost all be attributed to alteration of the actin-based cytoskeleton. These pathways are anchored around RhoA (ras homolog gene family, member A) and ILK (integrin-linked kinase) signaling (Fig. 1C). The Rho family of proteins (14 members) are guanosine triphosphatases. They are crucial for remodeling of the actin cytoskeleton^28^. ILK-signaling is not only known to mediating cell adhesion and interaction with the extracellular matrix but also for the orderly accumulation of F-actin inside of the cell membrane at around the sites of integrin attachment and the formation of stress fibers and focal adhesion^29^. Indeed, 33 from among the 98 DEGs (e.g. 33.7%) regulated only through the *S*. *aureus* infection were factors related to cytoskeletal structures or their regulators (Supplementary Table 3). In contrast, *E*. *coli* infection modulated only the expression of ACTC1 (actin, alpha, cardiac muscle 1) from among all of the cytoskeleton related factors.

### Different upstream regulators governed the udder response towards *E*. *coli* or *S*. *aureus*

**Figure 2:**
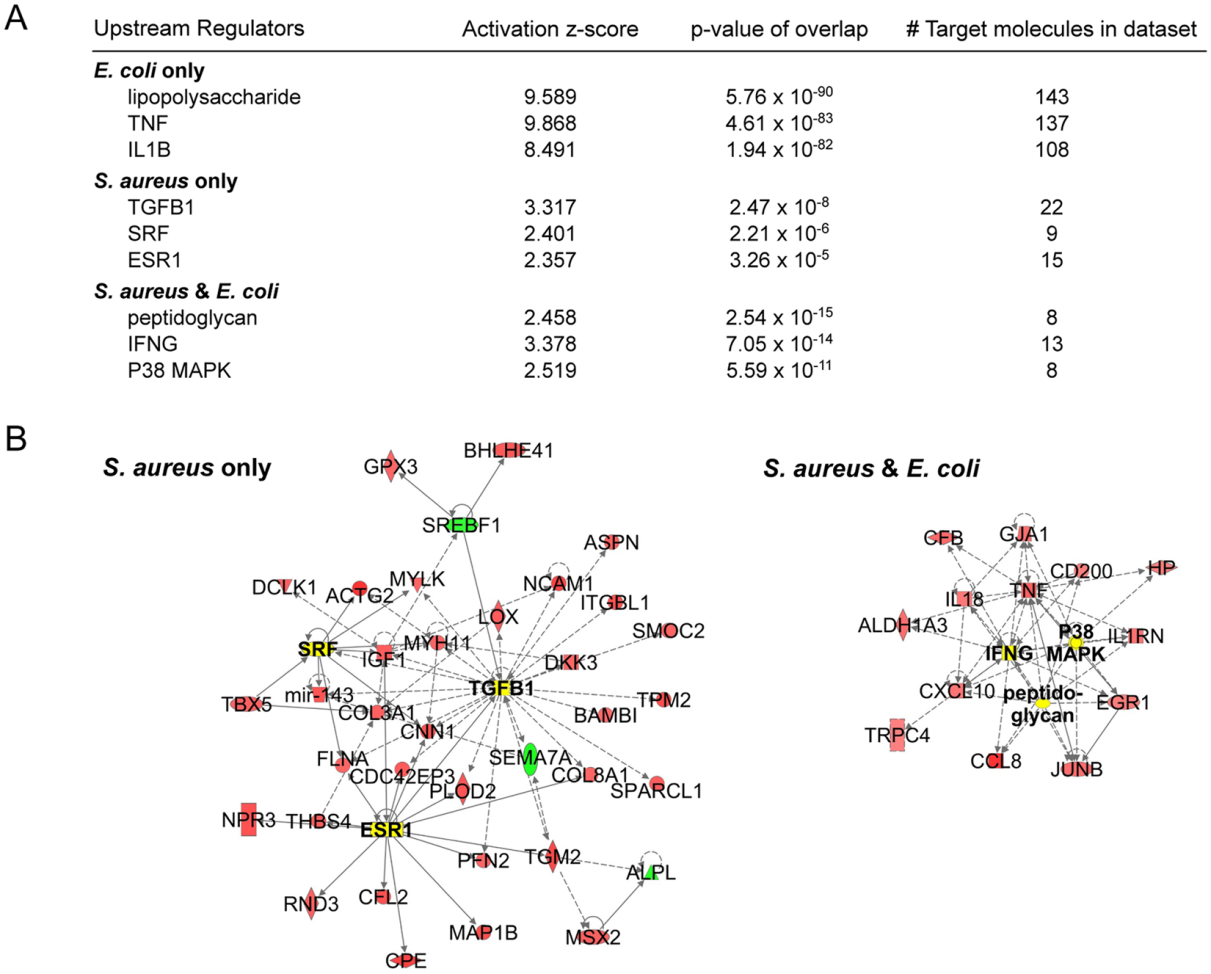
Ingenuity based identification of Upstream Regulators. (**A**) List and statistical significance of the 3 top-scoring upstream regulators as identified exclusively after *E*. *coli* or *S*. *aureus* infection or being commonly active during infection with both pathogens. (**B**) Graphical display of the regulatory networks governing the response towards an *S*. *aureus* infection (left) or that with both pathogens (right). Red and green underlay indicate up- or downregulation, respectively, while yellow highlights the up-stream regulators. Figure S2 shows the graphical display of the highly complex regulatory network during an *E*. *coli* infection.

We exploited the Ingenuity pathway analysis program to interrogate the lists of DEGs regarding key upstream regulators of the complex patterns of the pathogen-specific immune responses of the udders. Considering only the 3 top scoring potential regulators, we found (Fig. 2A) that the udder response towards the *E*. *coli* infection was guided by the effects elicited through the bacterial cell wall component LPS (lipopolysaccharide) and the well-known master cytokines TNF (tumor necrosis factor α) and IL1B (interleukin 1B). More than 100 of the *E*. *coli* specific DEGs were found to be associated with gene regulatory networks being governed by anyone of these 3 upstream regulators. The genes involved are listed in the Supplementary Table 3A. The graphical representation of the multitude of their regulatory interdependence can only superficially visualize the high complexity of those networks (Supplementary Figure 2). Abundance of the chemical compound LPS has not been measured, but *E*. *coli* infection had increased the mRNA concentrations encoding TNF by 8 fold and IL1B by almost 14 fold (Supplementary Table 3). These data show that inducing inflammation characterized the very early response of the udder after an infection with *E*. *coli*.

The regulatory networks of those genes having exclusively been regulated during the infection with *S*. *aureus* were less complex (Fig. 2). The networks activated by the top ranking upstream regulators TGFB1 (transforming growth factor beta 1), SRF (serum response factor) and ESR1 (estrogen receptor 1) comprised only 22 DEGs or less. The expression of none of these 3 master regulators was found to be regulated in our samples. However, the high statistical significance attributed to these pathways indicates that the expression of a considerable fraction of their downstream factors was modulated by the *S*. *aureus* infection (Fig. 2A).

The 16 DEGs regulated in common during infection with either of the pathogens are known to belong to networks being regulated by peptidoglycan, interferon γ (IFNG) or the mitogen activated kinase p38 (P38MAPK). We did not measure the abundance of peptidoglycan. The mRNA concentration of the MAP3K8-encoding gene-a representative of the MAP kinases-was 3.6 fold upregulated in the *E*. *coli* infected udders (at 3 h pi) while that of IFNG had not been changed during the udder infections. However, the relevance of all these 3 upstream regulators during mastitis is well known (see below).

### *E*. *coli* and *S*. *aureus* infection induced different immune dampeners

The strong inflammation having been induced by the *E*. *coli* infection was counter-acted by the induction of a broad spectrum of immune-dampening factors. Such factors having exclusively been induced by the *E*. *coli* infection included several TNFα induced proteins (TNFAIP2^30^; TNFAIP3^31^, also known as ubiquitin-editing enzyme A20; TNFAIP6^32^) the suppressors of cytokine signaling SOCS1, SOCS3^33^ and the NF-κB inhibitors NFKBIA, NFKBID, NFKBIE^34^. Also the dampener of inflammasome formation mir223^35^ and the inhibitor of NF-κB p65 phosphorylation NR4A1^36^ have only been induced during the *E*. *coli* infection. Induction of their expression can collectively be explained by the action of those upstream regulators.

*S*. *aureus* infection had also triggered the expression of several factors counteracting inflammation. However, only two of them (IL1RN, CD200^37^) had also been induced by the *E*. *coli* infection. *S*. *aureus* infection exclusively increased the levels of mRNAs encoding SLIT2 (slit homolog 2 protein), the micro-RNA miR-143 and C1QTNF3 (C1q and tumor necrosis factor related protein 3), all known to counteracting LPS induced inflammation. SLIT2 inhibits NF-κB activation^38^, miR-143 downregulates TLR2 expression^39^ while C1QTNF3 is a direct antagonist of TLR4 signaling by prohibiting binding of its ligand LPS^40^. RSPO3 (R-spondin-3)^41^ and CTHRC1 (collagen triple helix repeat containing 1)^42^ complete the list of inhibitory factors having exclusively been induced during the *S*. *aureus* infection. Both factors are activators of the wnt/β-catenin-signaling pathway which, in turn is known to inhibit inflammation through confining NF-κB activation by the stabilizing the NF-κB inhibitory IκB-factors^43^.

### *S*. *aureus*, but not *E*. *coli* swiftly invaded MEC

The clear indication that *S*. *aureus*, but not *E*. *coli* infection triggered cytoskeletal rearrangement in the host cells shortly after udder infection could relate to the known high potential of *S*. *aureus* pathogens to invade udder cells. Hence, we examined if the invasion potential of the *E*. *coli*_1303_ and *S*. *aureus*_1027_ strains having been used for infection would be significantly different. We therefore co-cultured the mammary epithelial model cell MAC-T for 20 min or 1 h with live *E*. *coli*_1303_ or *S*. *aureus*_1027_ at a MOI of 10 and determined the fraction of internalized pathogens. Only a very small fraction of the *E*. *coli* pathogens was internalized after 1 h of co-culture (<1% of the inoculum) while almost 20% of the *S*. *aureus* pathogens was internalized after that time (Table 3). Indeed, already after 20 min of co-culture had 3% of the *S*. *aureus*_1027_ inoculum been internalized, while no internalized *E*. *coli*_1303_ pathogens could be detected after this short time of co-culture.

**Table 3:** Invasion of pathogens into MAC-T cells.

Pathogen	strain	Attributed phenotype	Co-culture time		N^a^
			20 min	60 min	
*S*. *aureus*	1027	clinical^b^	3.0 ± 1.4	12.3 ± 6.6	4
	RF122	clinical^b^	0.7 ± 0.4	13.0 ± 0.7	2
	B	sub-clinical^b^	0.6	3.8	1
	D	sub-clinical^b^	6.2	13.4	1
*E*. *coli*	1303	clinical^b^	0.00	0.47 ± 0.27	2
	ECC-Z	persistent^c^	0.02 ± 0.003	0.045 ± 0.02	3
	ECA-0157	transient^c^	0.007 ± 0.001	0.02 ± 0.005	2
	ECA-727	transient^c^	0.006 ± 0.001	0.02 ± 0.004	2

Values represent mean percentages (±SEM) of the inoculum found inside the cells after co-culturing pathogen and host cells for the times as indicated. ^a^Number of independent experiments, each assayed in duplicate. ^b^Originally isolated from clinical or sub-clinical cases of mastitis, *cf* ref. 65; ^c^was shown to cause transient or persistent infection of udder cells, *cf* ref. 44.

We compared these values to those collected from 3 more strains each of *E*. *coli* and *S*. *aureus* mastitis pathogens and found them to be representative. The efficiency of all *S*. *aureus* strains examined to invaded the MAC-T cells was higher by at least 1 or 2 orders of magnitude (internalization of approximately 10–40% of the inoculum) than that of all those *E*. *coli* strains (0.02–0.05% internalization; Table 3). The latter data are quantitatively very similar to the previously reported potential of the same *E*. *coli* strains (ECC-Z, ECA-0157, ECA-727) to invading MAC-T cells^44^. Re-calculation of the data from that report shows that no more than 0.06% of the inoculum of any of these strains had invaded the host cells after 1 h of co-culture. Hence, all the *S*. *aureus* strains very clearly invaded the MEC host cells while the capacity of *E*. *coli* strains to invade the host cells was much lower.

### *S*. *aureus* infection modulated the actin-dependent cytoskeleton

**Figure 3:**
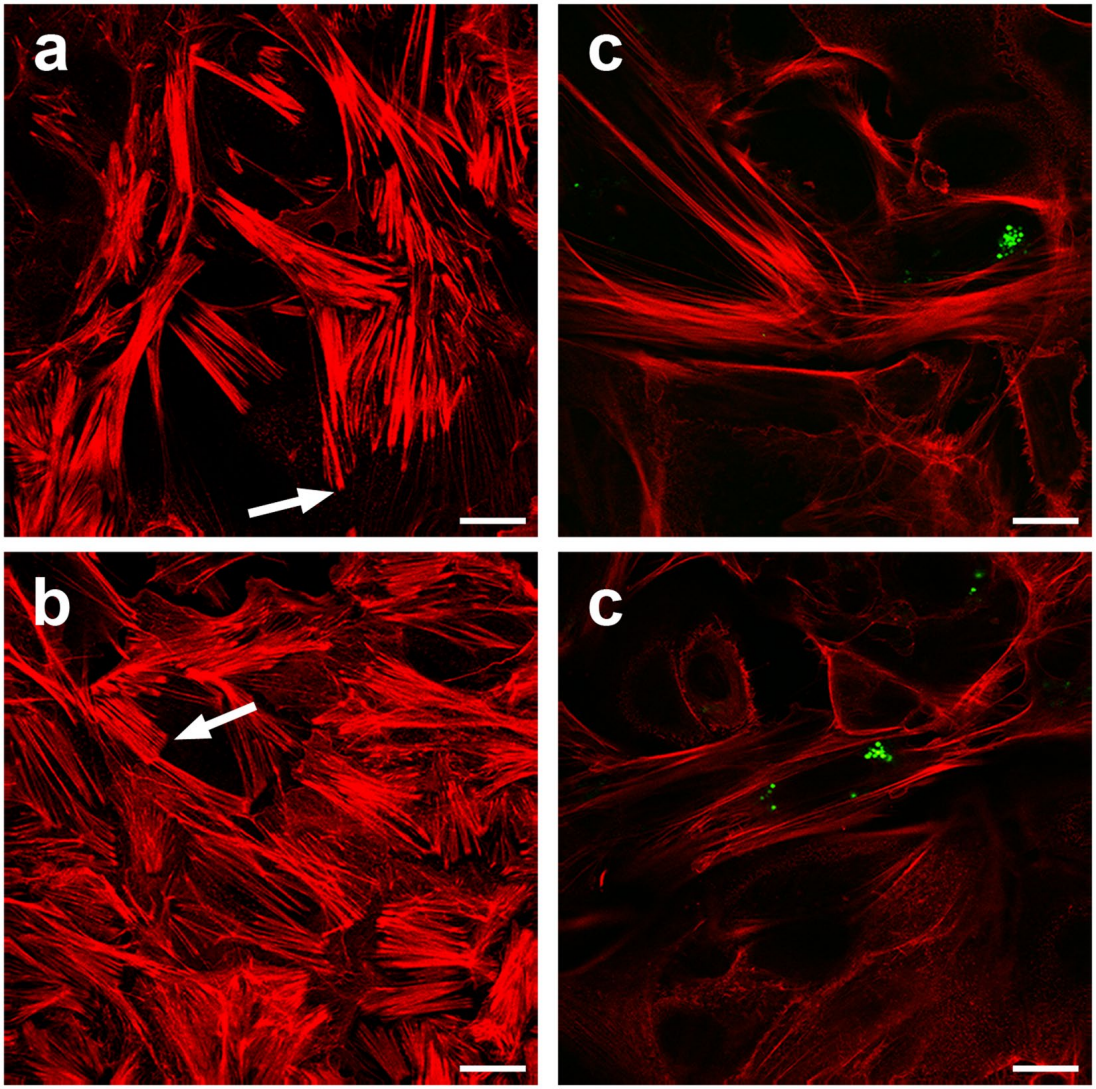
*S*. *aureus* invasion alters structure of the actin-cytoskeleton. Actin-fibers were stained with Alexa-Fluor tagged phalloidin from (**a**) control pbMEC cultures or (**b**) after 1 h co-culture with live *E*. *coli*_1303_ or (**c**) GFP-tagged *S*. *aureus*_1027_ pathogens. Images from control and *E*. *coli* co-cultured cells were dominated by short, thick ventral actin-stress fibers, mostly terminated by knob-forming focal adhesion foci. These were not evident in cells harboring the green fluorescent *S*. *aureus* pathogens. Instead, the actin fibers appeared more filamentous without apparent adhesion foci. (Scale bar, 10 µm).

We examined if *S*. *aureus* internalization would structurally alter the actin-dependent cytoskeleton. We therefore stained actin fibers with fluorescent-labeled phalloidin in primary MEC cells having either been co-cultured for 1 h with GFP-tagged *S*. *aureus*_1027–158_ or un-tagged *E*. *coli*_1303_ and in untreated control cells. Laser-scanner images revealed no difference in the appearance of the actin-stress fibers in control and *E*. *coli* treated cells. These images dominantly revealed thick actin bundles in both ventral as well as transversal fibers (Fig. 3). The ventral fibers were very clearly terminated at either side by knobbed actin aggregates, conceivably indicating focal adhesion foci. *S*. *aureus* harboring cells however revealed finer and longer actin fibrils which had not so evidently been terminated by prominent focal adhesion points. These differences become quite apparent by consecutively running through optical serial sections from the ventral to the dorsal side of the cells (Supplementary video). Such serial sections reveal in particular thin dorsal fibers in the *S*. *aureus* infected cells but not in their adjacent uninfected neighbors. While these images are intriguing, it is clear that more and different experiments would be required to quantify and eventually elucidate the significance of these structural alterations.

## Discussion

To the best of our knowledge, this is the first study globally profiling the very first transcriptional changes discriminating the *E*. *coli* vs *S*. *aureus* infection elicited immune responses of the udder. Hence, it gives an account of the pathogen-specific activation of innate immune mechanisms in the udder. It complements our initial description of the infection model reporting also the modulated expression of a limited set of candidate genes^24^. Our key result is that, in the absence of any recognizable systemic influences or cross-talk between infected and uninfected udder quarters, both pathogen species switched the udder response into entirely different, in some aspects opposite directions. *E*. *coli* infection triggered a strong inflammatory response, already as early as 2 h after infection. *S*. *aureus* failed to substantially induce those inflammatory pathways but instead triggered very significantly rearrangements of the cytoskeleton and some immunosuppressive mechanisms. The latter two observations are entirely novel, indeed.

### Results may be representative for the immune reaction of the udder against a broader spectrum of *S*. *aureus* pathogens

The current study was based on samples from the gland cistern (GC). Relevance of the distal udder compartments (including GC) for triggering the early immune response has already previously been highlighted by others^23, 45^: It was found during udder infection with *E*. *coli* lasting for 12 h or 24 h that the immune response in this tissue occurred somewhat faster than in the lobulo-alveolar tissue but that in principle the same regulatory networks of immune gene expression had been triggered in both tissues^45^. Similarly, long lasting udder infections with *S*. *aureus* (2–30 days) induced in both these tissues the expression of key cytokines, pathogen receptors and acute phase response factors in similar directions, albeit to different extents^23^. The lobulo-alveolar tissue comprises the mammary epithelial cells and the pathogen specific reaction norm of this cell type governs the immune response of the udder^11^. Hence, the immune response pattern of the GC tissue allows predicting with some confidence the overall pathogen-specific immune response of the udder.

We have only used a single *S*. *aureus* strain for udder infections. However, different commonalities of *S*. *aureus*_1027_ with other *S*. *aureus* strains together suggest that our results may be representative for a wider spectrum of *S*. *aureus* strains. We have seen in many experimental udder infections with this particular strain *S*. *aureus*_1027_ that it elicited in >90% of the cases (35/38; W. Petzl, unpublished observation) a weak and subclinical mastitis just as it is commonly observed in *S*. *aureus* caused mastitis. Moreover, this strain shares with other widely used *S*. *aureus* strains (N305 Niewbold; RF122) the failure to activate in MEC model cells the IκB/NF-κB signaling cascade^46^, representing a key lesion in the activation of immune functions. Last, we have seen in the present study that *S*. *aureus*_1027_ can invade MEC model cells just as well as other *S*. *aureus* strains.

### *E*. *coli* infection drives inflammation through TLR-signaling

It is long known that *E*. *coli* infection of the udder swiftly induces a strong inflammatory response^3, 19^ triggered by the activation of a battery of pathogen receptors, including members of the TLR-family and other pathogen recognition receptors (PRRs)^2, 13, 17, 19, 20^. Our current data fully comply with the previous findings. The list of the *E*. *coli* infection specific DEGs shows the typical vigorous induction of cytokine gene expression^6^ triggered through a prominent activation of the TLR signaling cascade. The latter is revealed by the upregulated expression of TLR2, for example and the modulated expression of several NF-κB factors and their inhibitors. All TLRs (except TLR3) and the NOD family of pathogen receptors are known to ultimately activate the NF-κB complex of transcription factors^47^. These factors are master regulators of immune functions known to regulating a wealth of immune genes^18^. The knowledge based bioinformatics analysis highlighted LPS (the authentic ligand for TLR4), TNF and IL1 as upstream key regulators governing the very early immune response in the udder. Also this aspect is entirely consistent with our current knowledge regarding *E*. *coli* mastitis^2^. Hence, our transcriptome data as related to the *E*. *coli* infection recapitulate that the key features of the *E*. *coli* specific immune response of the udder are dominated by innate immune functions of the mammary epithelial cells^11^ residing in the GC. Our data add as new information that this response was triggered without a significant contribution of a generalized systemic response. However, the increased IL10 mRNA concentration in the GC tissue at 3 h pi shows that the response of some professional immune cells had been triggered, either from those residing in the udder or having already been recruited into the gland. Cells other than mammary epithelial cells, such as macrophages are the source of IL10 in the udder^11^.

### *S*. *aureus* infection does not trigger classical PRR-signaling and NF-κB activation in the gland cistern

*S*. *aureus* infection did not modulate the expression level of any of the NF-κB factors or their inhibitory IκB regulators. It has frequently been observed in MEC that *S*. *aureus* infection or stimulation does not lead to an activation of the NF-κB factor complex^11, 13, 17, 46^ albeit it does so in professional immune cells^11, 13, 46^. Our study now validates that NF-κB activation is lacking *in vivo* in the GC tissue of the udder during an infection with live *S*. *aureus* pathogens indicating the lack of any productive TLR-signaling. We recognize the failure to activating the TLR signaling cascade in the udder during an *S*. *aureus* infection as a first crucial determinant discriminating *S*. *aureus* from *E*. *coli* mastitis. It explains in part the notorious belated and low intensity immune response of this gland to that pathogen species^2, 6^.

### Upstream regulators indicate activation of immune dampening quickly after *S*. *aureus* infection

We have identified TGFB1, ESR1 and SFR as upstream regulators dominating the response of the GC tissue against the *S*. *aureus* infection. Considering the immune dampening properties of these factors we note that the immune modulating effects of the pleiotropic cytokine TGFB1 have in many instances-albeit not exclusively-been shown to be immune suppressive^48^. The immune dampening properties of this factor rely in part on down-regulating the MyD88 dependent branch of TLR-signaling through destabilizing the MyD88 protein rather than reducing its mRNA concentration^49^. Hence, high threshold levels of TGFB1 in epithelial cells^50^ constantly confine TLR2 or TLR4 signaling, as clearly evidenced by the endotoxin hypersensitivity of TGFB1 null model mice^51^. We did not record any modulation of the TGFB1 mRNA levels during our short term infection in this study. However, the TGFB1 factor is secreted as an inactive pro-factor requiring proteolytic activation^52^, a process which we did not monitor in the current study. Moreover, it was recently shown in longer term experimental mastitis trials of cows that the un-infected healthy control cows featured high TGFB1 mRNA levels and these were maintained during massive *S*. *aureus* infection of the udder, whilst the TGFB1 mRNA levels were down-regulated in the udder during *E*. *coli* mastitis^4^. In support, prolonged (>48 h) *S*. *aureus* infection of the udder increases the concentration of TGFβ1 in the milk^53^.

Regarding the immune dampening properties of both other upstream regulators, it is well known that ESR1 mediated effects of estrogen are often immune suppressive^54^. SRF, signaling through the co-factor ELK4 may be immune suppressive as well, since this signaling pathway induces IL10^55^ and ERG1 expression^56^. ERG1, in turn is long known to directly stimulate TGFB1 expression^57^.

### Pathogen-dependent differentiation in the activation of mechanisms attenuating the immune response

The comparison of all the immune dampening features as they had been induced by either of the pathogen species reveals a pathogen-specific differentiation. *E*. *coli* induces a battery of countermeasures to directly dampen cytokine- and pathogen-signaling. The three dominant upstream regulators of the response against *E*. *coli* (LPS, IL1, TNF) have in common that they are all activating the IκB/NF-κB signaling cascade (GO ID:0007249). LPS-signaling through TLR4-and IL1-signaling through IL1 receptor-will both activate their TIR-domains and thereby trigger NF-κB activation^46^. TNF-binding to a member of the tumor necrosis factor superfamily of receptors-activates the intracellular death domain (DD) of the receptor and thereby also ultimately activates NF-κB^58^. Hence, it is not surprising to find that most of the *E*. *coli* infection specific dampeners of immune activation are interfering with the IκB/NFκB-dependent downstream signaling of those cytokine receptors and PRRs. They establish negative feedback loops of their signaling and these all are operating in the cytoplasm of the target cell. However, these immune dampening mechanisms are juxtaposed and overwhelmed by a plethora of factors and pathways promoting inflammation. Consequently, inflammation prevails during early *E*. *coli* mastitis.

**Figure 4:**
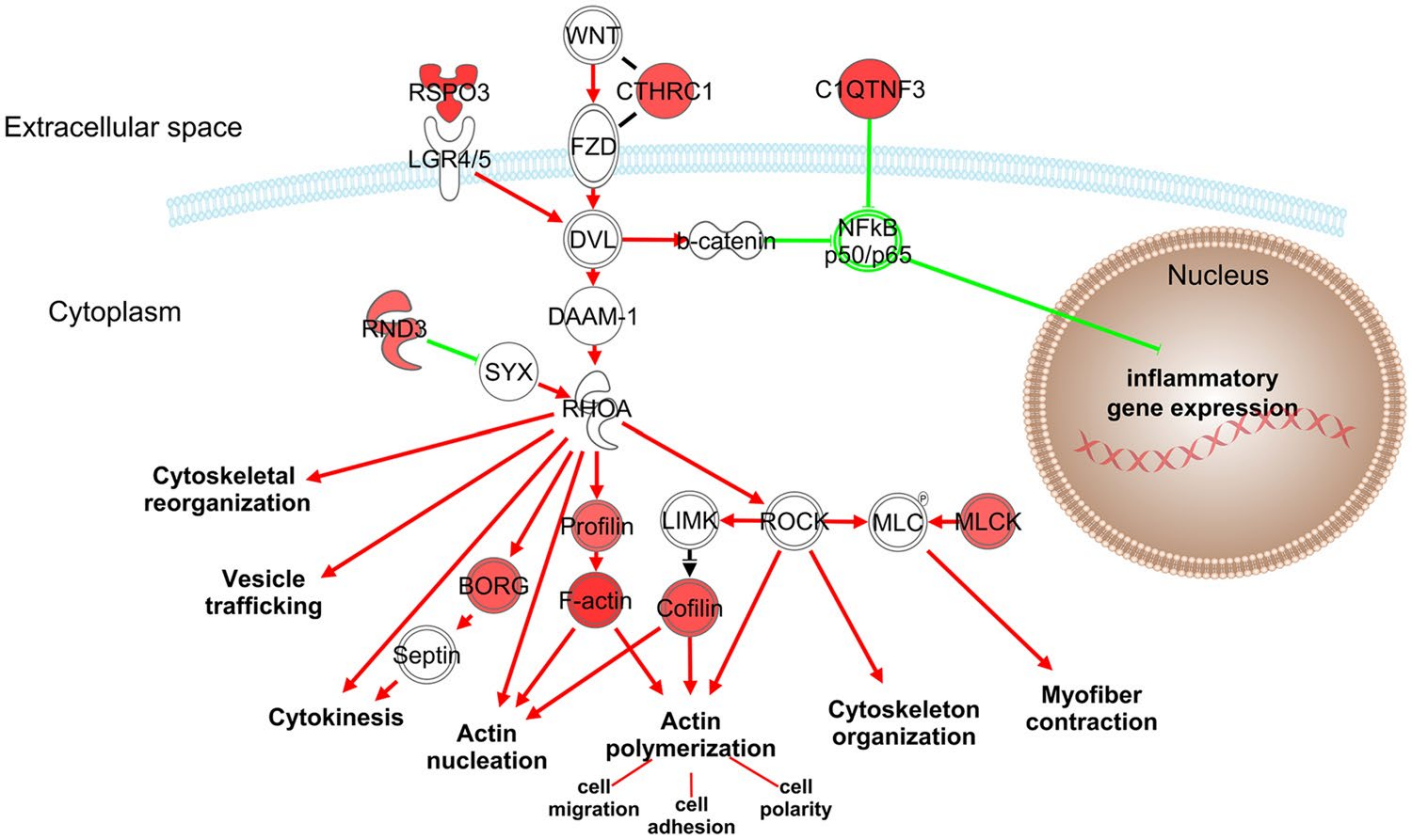
Summary of the regulatory events triggered by the *S*. *aureus* infection. Red and green colors symbolize activation or repression, respectively. BORG: binder of Rho GTPases (here: the DEG CDC42EP3); C1QTNF3, C1q and tumor necrosis factor related protein 3; CTHRC1, collagen triple helix repeat containing 1; DAAM-1, disheveled associated activator of morphogenesis 1; DVL, disheveled; FZD, frizzled receptor; LGR4/5, leucine-rich repeat-containing G-protein coupled receptor 4/5; LIMK, Lim kinase; MLC: myosin light chain; MLCK, myosin light chain kinase; RHOA, ras homolog family member A; RND3, Rho family GTPase 3; ROCK, Rho kinase; RSPO3, r-spondin 3; SYX, pleckstrin homology and RhoGEF domain containing G5.

*S*. *aureus*, in contrast triggers immune dampening pathways swiftly after infection which are unrelated to cytokine- and PRR-signaling. This reflects and underscores the fact that these receptors are not activated by that pathogen in the epithelial cells. These immune dampeners may either directly down-regulate PRR abundance (miR-143 related destruction of TLR2 mRNA) or quench the immune responsiveness through enhanced secretion of the immune suppressive factors RSPO3, CTHRC1 and C1QTNF3 (Fig. 4). These factors (i) not only trigger immune suppression through mechanisms being completely unrelated to those as triggered by *E*. *coli* infection, but (ii) their functioning is not restricted to the target cell. Rather, these factors will also repress the immune responsiveness of surrounding cells. Most significant for the physiology of *S*. *aureus* mastitis is the fact that their effects are not outweighed by a strong stimulation of inflammation due to diminished cytokine- and absent PRR-signaling. Hence, a key aspect of *S*. *aureus* mastitis is unbalanced activation of immune dampening mechanisms and this represents a second major discriminator from *E*. *coli* mastitis.

### Rearrangement of the cytoskeleton in the host cells is a dominant feature of *S*. *aureus* infection

Considering the actin-cytoskeleton modulating properties of the *S*. *aureus* infection-specific upstream regulators, it has been shown that SRF, signaling through the co-factors MKL1/2 (myocardin-related transcription factors) is a key regulator of cytoskeletal rearrangements in immune cells^56^. This aspect is fully supported by the current analysis of the key signaling pathways activated by the *S*. *aureus* infection and uncovering them provides deeper insights into the responsible mechanisms. Regarding rearrangement of the actin-dependent cytoskeleton, it is known that the activity of the RhoA GTPases is of pivotal importance for regulating the structure of the actin-dependent cytoskeleton^28^. Pathogenic bacteria developed different strategies to facilitate invasion into the host cell through manipulating the activity of Rho GTPases^59^. *S*. *aureus* may adhere to endothelial cells *via* the fibronectin binding protein^60^. This induces local β1-integrin aggregation triggering reorganization of the actin cytoskeleton *via* Rho GTPase activation and this allows for *S*. *aureus* invasion^61^. Augmenting this, we identified signaling through the integrin-linked kinase (ILK) also as a major player of the host defense in response to the *S*. *aureus* infection. This kinase is involved in the control of actin accumulation and rearrangement of the cytoskeleton^29^. Hence, the bioinformatics analysis of the transcriptome data lines up well with our respective experimental data. They together reveal very clearly that *S*. *aureus* induces quickly after infection rearrangement of the actin cytoskeleton and conceivably exploits it to invade the epithelial cells of the host. In contrast, *E*. *coli* infection did not induce any of these signaling pathways or structural alterations. Moreover, our comparison of the differential capacity of *E*. *coli* and *S*. *aureus* pathogens to invade the MAC-T model cell for MEC clearly validated the long known facts that (i) *E*. *coli* has a much weaker potential to invade those cells than enteric bacterial pathogen species^62^ and (ii) that many *S*. *aureus* strains have a genuine capacity to invading MEC^63^. Hence, invasion of *S*. *aureus* into the host cells very quickly after infection is a third crucial discriminator between *S*. *aureus* and *E*. *coli* mastitis.

In summary, our data regarding the *S*. *aureus* specific features of the very early events after infecting the gland cistern show that the expression of three extracellular factors (C1QTNF3, CTHRC1, RSPO3) is enhanced (Fig. 4). They inhibit inflammation through blocking NF-κB activation, either directly through prohibiting pathogen recognition (C1QTNF3) or through stimulating the wnt/β-catenin signaling cascade (CTHRC1, RSPO3). The latter will modulate the activity of the Rho GTPases which, in turn will lead to rearranging the actin-cytoskeleton facilitating invasion of *S*. *aureus* into the epidermal cells of the host.

**Figure 5:**
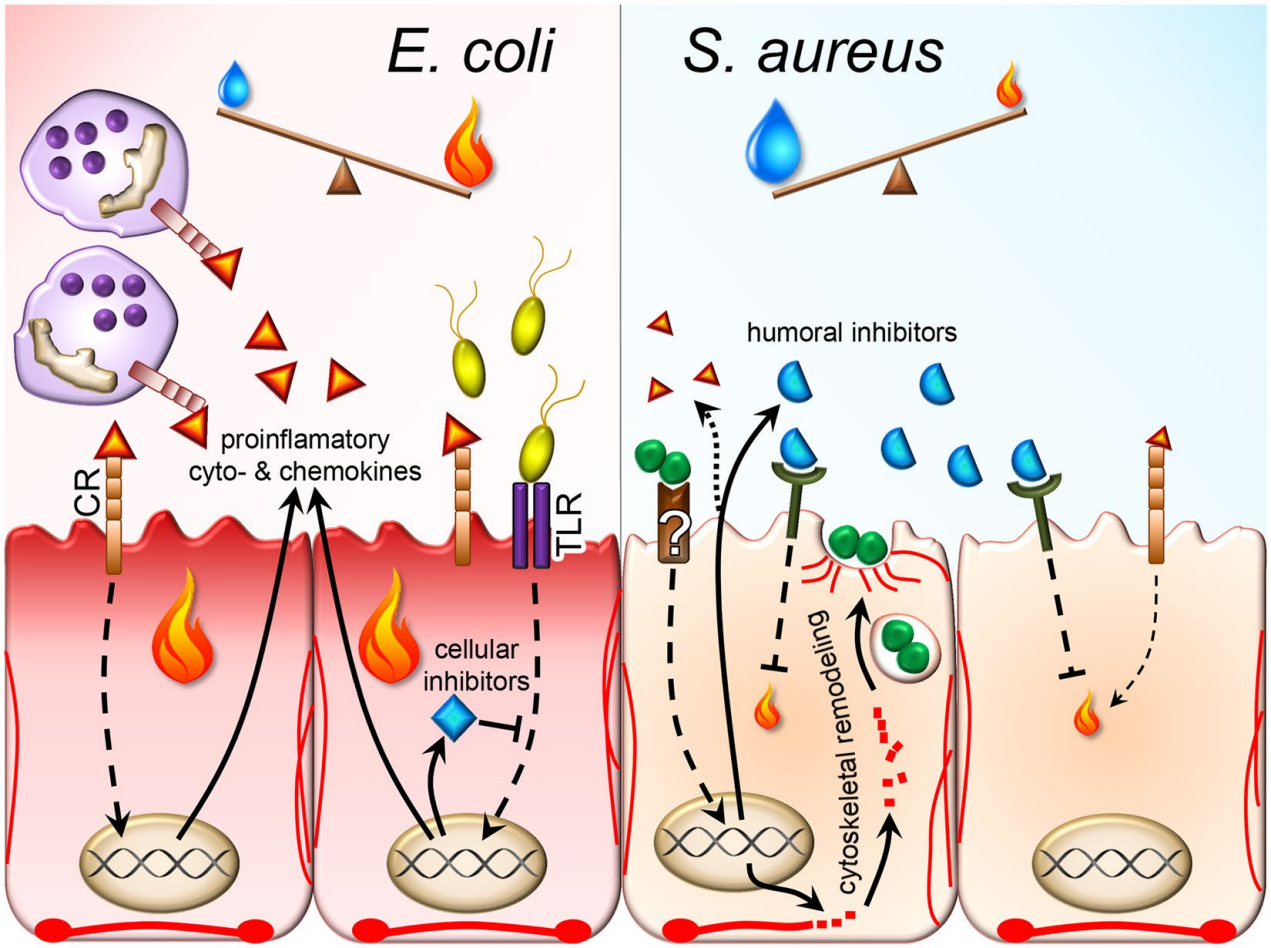
Graphical summary of the key differences between the early events after *E*. *coli* vs *S*. *aureus* udder infection. *E*. *coli* (yellow) activates in the epithelial cells TLR signaling triggering synthesis and secretion of proinflammatory factors (cyto- and chemokines, triangles), recruiting and activating cellular factors of innate immunity through activating cytokine receptor (CR) signaling. Also immune dampening factors are activated (blue diamond) but, on balance inflammation (flame) outweighs immune dampening (blue water drop). Knobbed red lines symbolize the ventral fibers terminated by focal adhesion plaques. No alteration of the actin cytoskeleton occurs. *S*. *aureus* (green Staphylococci) presence is perceived by the epithelial cell through unknown receptors rather than through TLRs or other classical PRRs. This triggers synthesis and secretion of immune dampening factors (blue crescents) surmounting in their effect any proinflammatory stimulation. Moreover, *S*. *aureus* is internalized into the epithelial cells, facilitated through rearrangement and interaction with the actin cytoskeleton. This enables pathogen persistence eventually resulting in chronic infection.

Taken together, we have first time deciphered the fundamentally different host response patterns triggered in the udder quickly after an *E*. *coli* or *S*. *aureus* infection (Fig. 5).

I. We recognize as a first crucial determinant discriminating *S*. *aureus* from *E*. *coli* mastitis the failure to activating PRR signaling cascades in the udder during an *S*. *aureus* infection. This results in a diminished inflammatory response which is not strong enough to outweigh immune dampening mechanisms.
II. Hence, unbalanced activation of immune dampening mechanisms during *S*. *aureus* mastitis represents a second major discriminator from *E*. *coli* mastitis.
III. The capacity of *S*. *aureus* to invading the epithelial cells of the host very quickly after infection is a third crucial discriminator between *S*. *aureus* and *E*. *coli* mastitis.

## Material and Methods

### Bacterial strains

The bacterial strains used for udder infusion (*E*. *coli*_1303_^64^ and *S*. *aureus*_1027_) have originally been isolated from clinical cases of mastitis and their cultivation and asseveration has been described^19^. We have used them ever since in many experimental udder infections of healthy first lactating heifers. That particular *E*. *coli* strain 1303 had always elicited severe clinical mastitis within 24 h after infusing 500 CFU into a single udder quarter (n, 38). Infusing 10,000 CFU of *S*. *aureus*_1027_ had caused subclinical mastitis in 35 cows while causing severe mastitis in 3 cows. That *S*. *aureus* strain has in addition been characterized at the genomic and proteomic level^65^ together with the *S*. *aureus* strains RF122 (originally isolated from clinical mastitis), B and D. The latter two strains had been isolated from subclinical cases of mastitis. Prof. Susanne Engelmann kindly provided the green fluorescent protein (GFP) expressing strain *S*. *aureus*_1027–158_ having been established by her through transforming *S*. *aureus*_1027_ with the plasmid pMV158gfp^66^. *E*. *coli* strains ECC-Z, ECA-0157 and ECA-727 have also previously been described^67^. Strain Z had been classified as causing persistent infections of MEC model cells, while both others were classified as causing transient infections. These strains have kindly been provided by Prof. Ynte Schukken.

### Experimental setting and samples

The study was designed to compare and discriminate the earliest modulations of the transcriptome of the udder in response to *E*. *coli*_1303_ or *S*. *aureus*_1027_ infection based on global transcriptome profiling. It complements our initial description of the infection model including its clinical aspects, sample preparation and the modulated expression of a limited set of candidate genes^24^. The infection model featured infusing sequentially a high number (5 × 10^6^ CFU/udder quarter) of either *E*. *coli*_1303_ or *S*. *aureus*_1027_ pathogens into three of four udder quarter of healthy HF heifers at mid lactation (n = 6 animals for *E*. *coli* and *S*. *aureus*, respectively). The trial started by infusing the pathogens into the left hind quarter. The left front and right hind quarters were infused 1 h and 2 h later, respectively. The right front quarter remained untreated and served as baseline control. Cows were culled at 3 h pi, hence well before any clinical symptoms (fever, leukopenia, clots in milk, increased counts of somatic milk cells counts [SSC], leukopenia) were detectable. Those parameters had been monitored at 1 h intervals. The fact that SSC values remained largely unchanged indicated by corollary absence of considerable cross-talk between infected and control quarters that shortly after infection. It has frequently been observed during longer lasting udder infections (24 h) with *E*. *coli*, but not with *S*. *aureus*, that the infection of a single udder quarter had increased the expression of immune genes (cytokines, bactericidal genes) in the neighboring uninfected control quarter^4, 20^. Increased gene expression in the controls causes in tendency to underestimate the infection related extent of induced immune gene expression during *E*. *coli* mastitis in such experimental settings. Immediately after culling (<5 min), samples from the teat cistern, gland cistern and milk-producing parenchyma were snap frozen in liquid nitrogen and stored herein. Based on that initial profiling of the udder responses we selected for the current study the samples collected from the gland cisterns (GC) of the infected udders. Infections with both pathogen species had induced in this compartment clear and significant changes in the expression profiles of our candidate genes while little changes had been recorded in proximal locations of the lobulo-alveolar milk-producing parenchyma.

### Ethics Statement

Our previous report about this infection trial^24^ included also the statement that the animal experimentation complied with the pertinent ethical standards as outlined in the German Tierschutzgesetz (TierSchG) and had been approved by the responsible ethics committee of the regional government of Upper Bavaria, Germany (No. 55.2-1-54-2531-102-09, issued at 21st September, 2009).

### Microarray Analysis

For profiling mRNA expression in the GC tissue, we used the custom Affymetrix® GeneChip® BovGene-1_0-st Bovine Gene 1.0 ST Array (GPL16500, Affymetrix, Santa Clara, USA). The design of this Array was based on Ensemble and RefSeq predictions for the Genome Bos Taurus Built 4.0. In total, the array contains 194,712 probe sets representing almost 24,000 bovine transcripts. For hybridization, 500 ng of total RNA having been extracted with the RNeasy kit (Qiagen, Hilden, Germany) were amplified with the Ambion® WT Expression Kit (Ambion, Grand Island, NY, USA). Samples containing 5.5 μg of the resultant cDNA were fragmented, labelled (Affymetrix® GeneChip® WT Terminal Labelling Kit) and subsequently hybridized to the microarray using the Affymetrix® GeneChip® WT Hybridization Wash and Stain Kit following the pertinent Affymetrix standard protocols. The fluidic station protocol FS540_0001 was used. Slides were scanned with the GeneChip® Scanner 3000 7 G system (Affymetrix, Santa Clara, USA). Quality of the data was controlled with the Expression Console 1.2 software (Affymetrix, Santa Clara, USA) in accordance with the pertinent Affymetrix technical note (Affymetrix 2007). Background was adjusted with the robust multi-array average (RMA) algorithm which also served for quantile normalization, and summarization. Transcripts with significant (P < 0.05) expression levels were identified with the ‘detection above background’ (DABG) algorithm. We defined a gene as being expressed in our samples if (a) more than 50 percent of the exons were called as ‘Present’ and (b) the gene was called ‘Present’ in more than 50 percent of the samples within a group (Affymetrix technical note). The annotation used in this study was based on the UMD3.1 assembly of the cattle genome and only results from annotated transcripts have been considered in the bioinformatics analysis. The microarray data sets were submitted to the Gene Expression Omnibus (GEO) database (accession no. GSE94056). The Biometric Research Branch (BRB) array tools version 4.4.1 (http://linus.nci.nih.gov/BRB-ArrayTools.html) helped identifying differentially expressed transcript (DEGs). Applying the ‘class comparison between groups of arrays’ tool and using the ‘paired sample analysis’ function, we compared within the *E*. *coli* or *S*. *aureus* infection groups the expression levels of genes in the infected quarter at each individual time point to that of the uninfected control quarter of the same animal. Transcripts were defined as differentially expressed at the respective time point after infection if the mean fold change of the infected quarters relative to the unstimulated control quarters exceeded >1.5 and the p-value of the univariate t-test paired according the individual animal was <0.05. False discovery rates are listed in the Supplementary Table 1. This parameter was not applied as cut off criterion since it would have been too stringent for some comparisons, eventually resulting in no significantly regulated genes at all. The knowledge-based bioinformatics analysis of the DEGs was primarily based on the Ingenuity Pathway Analysis software (IPA, Quiagen, Redwood City, CA 94063, United States).

### Quantitative real-time PCR (RT-qPCR)

Preparation of cDNA from total RNA with Superscript II (Invitrogen, Karlsruhe, Germany) and mRNA copy number quantification using the LightCycler and the SYBR Green plus reagent (both Roche, Basel, Switzerland) was conducted just as previously described^10^. Sequences of the oligonucleotide primers are given in the Supplementary Table 2. Spearman rank correlations between microarray and RT-qPCR results were calculated with the GraphPad Prism Version 5 software package (GraphPad Software, Inc., La Jolla, CA, USA).

### Bacterial invasion assays

#### Quantitative assay

Pre-cultures were started by inoculating a single colony of the pathogen into 1.5 ml Brain Heart Infusion (BHI) broth and shaking the cultures overnight at 37 °C. Next, they were diluted 1:100 and grown at 37 °C on a shaker to a density of 0.5 at OD_600_ nm. Plating validated that this was equivalent to approximately 2.5 × 10^8^/ml of the *S*. *aureus*_1027_ and *E*. *coli*_1303_ pathogens.

We used the established MAC-T mammary epithelial model cell to analyze the invasiveness of *E*. *coli* and *S*. *aureus* pathogens. Maintenance cultures of MAC-T cells were cultivated at 37 °C in DMEM medium, supplemented with 10% FCS and 4 mM L-Glutamine as described^11^. For infection experiments, the medium was changed to HEPES (25 mM) buffered DMEM without antibiotics added. The cells were grown in 24 well plates to near confluency resulting in approximately 10^5^ cells/well. They were infected with 10^6^ pathogens, resulting in a multiplicity of infection (MOI) of 10. Pathogens were washed off with PBS (5x) after 20 min or 60 min and the cells were incubated in growth medium for another 2 h in the presence of 100 µg/ml of gentamycin to kill any external bacteria. Subsequently, cells were lysed for 20 min through the addition of an equal volume of medium containing 0.1% Triton X100. Dilutions hereof (1:10–1:1000) were plated to determine the number of internalized pathogens. Controls monitored the adherence of the bacteria to the plastic of the culture plates as well as external adherence to the MAC-T cells.

#### Qualitative assay

Primary bovine mammary epithelial cells (pbMEC) were prepared from udder tissue and cultivated RPMI 1640 medium on collagen type I coated tissue plates (Greiner bio-one) as described^9^. The strain *S*. *aureus*_1027–158_, expressing the green fluorescent protein (GFP) under the control of a tetracyclin stimulated promoter was grown up overnight at 37 °C, diluted by 1:100 and then grown up to an OD_600_ of 0.5 in tryptic soy broth supplemented with 10 µg/ml tetracycline. Bacteria were washed with-and re-suspended in-RPMI prior to applying them for infection. For conducting infection experiments, pbMEC were grown on collagen coated cover slips in HEPES buffered RPMI medium supplemented with 10 µg/ml tetracycline and infected with *S*. *aureus*_1027–158gfp_ at a MOI of 100 for 1 h. Subsequently, the cells were washed three times with PBS to remove any unbound *S*. *aureus*. The pbMEC were cultivated for another two hours in medium containing 5 µg/ml lysostaphin (Sigma Aldrich, prepared from *Staphylococcus staphylolyticus*) to lyse any external *S*. *aureus*. For control experiments, cover slips with pbMEC were left unstimulated or were similarly infected for 1 h with *E*. *coli*_1303_, washed with PBS and cultivated for another 2 h in RPMI medium. At the end of the experiment all cells were fixated for 10 min with 3.7% paraformaldehyde and permeabilized with 0.5% Triton X-100. Stress fibers were labelled with the red fluorescing ActinRed 555 ReadyProbes Reagent (ThermoFisher) following the standard protocol of the manufacturer. Finally, cover slips were mounted with DABCO antifading reagent (Carl Roth, Karlsruhe, Germany) and examined with a confocal laser scanning microscope (TCS SP2, Leica, Germany).

## Electronic supplementary material

Supplementary material

Supplementary video

## References

[1] Hogeveen H, Huijps K, Lam TJ (2011). Economic aspects of mastitis: new developments. N Z Vet J.

[2] Schukken YH (2011). Host-response patterns of intramammary infections in dairy cows. Vet Immunol Immunopathol.

[3] Burvenich C, Van Merrid V, Mehrzad J, ez-Fraile A, Duchateau L (2003). Severity of *E*. *coli* mastitis is mainly determined by cow factors. Vet Res.

[4] Jensen K (2013). *Escherichia coli*- and *Staphylococcus aureus*-induced mastitis differentially modulate transcriptional responses in neighbouring uninfected bovine mammary gland quarters. BMC Genomics.

[5] Almeida R, Dogan B, Klaessing S, Schukken Y, Oliver S (2011). Intracellular fate of strains of *Escherichia coli* isolated from dairy cows with acute or chronic mastitis. Vet Res Commun.

[6] Bannerman DD (2009). Pathogen-dependent induction of cytokines and other soluble inflammatory mediators during intramammary infection of dairy cows. J Anim Sci.

[7] Rinaldi M, Li RW, Capuco AV (2010). Mastitis associated transcriptomic disruptions in cattle. Vet Immunol Immunopathol.

[8] Hensen SM, Paviccaronicacute MJAM, Lohuis JACM, de Hoog JAM, Poutrel B (2000). Location of *Staphylococcus aureus* within the experimentally infected bovine udder and the expression of capsular polysaccharide type 5 *in situ*. J Dairy Sci.

[9] Günther J (2009). Assessment of the immune capacity of mammary epithelial cells: Comparison with mammary tissue after challenge with *Escherichia coli*. Vet Res.

[10] Goldammer T (2004). Mastitis increases mammary mRNA abundance of β-defensin 5, Toll-like-receptor 2 (TLR2), and TLR4 but not TLR9 in cattle. Clin Diagn Lab Immunol.

[11] Günther J, Koy M, Berthold A, Schuberth HJ, Seyfert HM (2016). Comparison of the pathogen species-specific immune response in udder derived cell types and their models. Vet Res.

[12] Lahouassa H, Moussay E, Rainard P, Riollet C (2007). Differential cytokine and chemokine responses of bovine mammary epithelial cells to *Staphylococcus aureus* and *Escherichia coli*. Cytokine.

[13] Yang W (2008). Bovine TLR2 and TLR4 properly transduce signals from *Staphylococcus aureus* and *E*. *coli*, but *S*. *aureus* fails to both activate NF-κB in mammary epithelial cells and to quickly induce TNFα and interleukin-8 (CXCL8) expression in the udder. Mol Immunol.

[14] Strandberg Y (2005). Lipopolysaccharide and lipoteichoic acid induce different innate immune responses in bovine mammary epithelial cells. Cytokine.

[15] Gilbert F (2013). Differential response of bovine mammary epithelial cells to *Staphylococcus aureus* or *Escherichia coli* agonists of the innate immune system. Vet Res.

[16] Brand B (2011). Comparative expression profiling of *E*. *coli* and *S*. *aureus* inoculated primary mammary gland cells sampled from cows with different genetic predispositions for somatic cell score. Genet Sel Evol.

[17] Günther J (2011). Comparative kinetics of *Escherichia coli*- and *Staphylococcus aureus*-specific activation of key immune pathways in mammary epithelial cells demonstrates that *S*. *aureus* elicits a delayed response dominated by Interleukin-6 (IL-6) but not by IL-1A or Tumor Necrosis Factor Alpha. Infect Immun.

[18] Hayden MS, Ghosh S (2011). NF-κB in immunobiology. Cell Res.

[19] Petzl W (2008). *Escherichia coli*, but not *Staphylococcus aureus* triggers an early increased expression of factors contributing to the innate immune defense in the udder of the cow. Vet Res.

[20] Mitterhuemer S (2010). *Escherichia coli* infection induces distinct local and systemic transcriptome responses in the mammary gland. BMC Genomics.

[21] Lutzow YC (2008). Identification of immune genes and proteins involved in the response of bovine mammary tissue to *Staphylococcus aureus* infection. BMC Vet Res.

[22] Brenaut P (2014). Contribution of mammary epithelial cells to the immune response during early stages of a bacterial infection to *Staphylococcus aureus*. Vet Res.

[23] Whelehan CJ, Meade KG, Eckersall PD, Young FJ, O’Farrelly C (2011). Experimental *Staphylococcus aureus* infection of the mammary gland induces region-specific changes in innate immune gene expression. Vet Immunol Immunopathol.

[24] Petzl W (2016). Early transcriptional events in the udder and teat after intra-mammary *Escherichia coli* and *Staphylococcus aureus* challenge. Innate Immun.

[25] Arend WP (1991). Interleukin 1 receptor antagonist. A new member of the interleukin 1 family. J Clin Invest.

[26] Weber NC, Blumenthal SB, Hartung T, Vollmar AM, Kiemer AK (2003). ANP inhibits TNF-α induced endothelial MCP-1 expression–involvement of p38 MAPK and MKP-1. J Leukoc Biol.

[27] Mao M (2004). T lymphocyte activation gene identification by coregulated expression on DNA microarrays. Genomics.

[28] Takai Y, Sasaki T, Matozaki T (2001). Small GTP-Binding Proteins. Physiol Rev.

[29] Sakai T (2003). Integrin-linked kinase (ILK) is required for polarizing the epiblast, cell adhesion, and controlling actin accumulation. Genes Dev.

[30] Thair SA (2016). TNFAIP2 inhibits early TNFα-induced NF-κB signaling and decreases survival in septic shock patients. J Innate Immun.

[31] Abbasi A, Forsberg K, Bischof F (2015). The role of the ubiquitin-editing enzyme A20 in diseases of the central nervous system and other pathological processes. Front Mol Neurosci.

[32] Choi H, Lee RH, Bazhanov N, Oh JY, Prockop DJ (2011). Anti-inflammatory protein TSG-6 secreted by activated MSCs attenuates zymosan-induced mouse peritonitis by decreasing TLR2/NF-kappaB signaling in resident macrophages. Blood.

[33] Mahony R, Ahmed S, Diskin C, Stevenson NJ (2016). SOCS3 revisited: a broad regulator of disease, now ready for therapeutic use?. Cell Mol Life Sci.

[34] Scheidereit C (2006). IkappaB kinase complexes: gateways to NF-kappaB activation and transcription. Oncogene.

[35] Haneklaus M, Gerlic M, O’Neill LA, Masters SL (2013). miR-223: infection, inflammation and cancer. J Intern Med.

[36] Ipseiz N (2014). The Nuclear Receptor Nr4a1 Mediates Anti-Inflammatory Effects of Apoptotic Cells. J Immunol.

[37] Rygiel TP (2009). Lack of CD200 Enhances Pathological T Cell Responses during Influenza Infection. J Immunol.

[38] Zhao H, Anand AR, Ganju RK (2014). Slit2–Robo4 pathway modulates lipopolysaccharide-induced endothelial inflammation and its expression is dysregulated during endotoxemia. J Immunol.

[39] Xia X (2016). Staphylococcal LTA-induced miR-143 inhibits *Propionibacterium acnes*-mediated inflammatory response in skin. J Invest Dermatol.

[40] Kopp A (2010). C1q/TNF-related protein-3 represents a novel and endogenous lipopolysaccharide antagonist of the adipose tissue. Endocrinol.

[41] Cruciat CM, Niehrs C (2013). Secreted and transmembrane wnt inhibitors and activators. Cold Spring Harb Perspect Biol.

[42] Kudryavtseva E, Forde TS, Pucker AD, Adarichev VA (2012). Wnt signaling genes of murine chromosome 15 are involved in gender-affected pathways of inflammatory arthritis. Arthritis Rheum.

[43] Silva-Garcia O, Valdez-Alarcon JJ, Baizabal-Aguirre VM (2014). The Wnt/beta-catenin signaling pathway controls the inflammatory response in infections caused by pathogenic bacteria. Mediators Inflamm.

[44] Dogan B (2006). Adherent and invasive *Escherichia coli* are associated with persistent bovine mastitis. Vet Microbiol.

[45] Rinaldi M (2010). A sentinel function for teat tissues in dairy cows: dominant innate immune response elements define early response to *E*. *coli* mastitis. Funct Integr Genomics.

[46] Bauer I, Günther J, Wheeler TT, Engelmann S, Seyfert HM (2015). Extracellular milieu grossly alters pathogen-specific immune response of mammary epithelial cells. BMC Vet Res.

[47] Kumar H, Kawai T, Akira S (2011). Pathogen recognition by the innate immune system. Int Rev Immunol.

[48] Wan YY, Flavell RA (2007). Yin-Yang functions of transforming growth factor-β and T regulatory cells in immune regulation. Immunol Rev.

[49] Naiki Y (2005). Transforming Growth Factor-β Differentially Inhibits MyD88-dependent, but Not TRAM- and TRIF-dependent, Lipopolysaccharide-induced TLR4 Signaling. J Biol Chem.

[50] Taylor AW (2009). Review of the activation of TGF-β in immunity. J Leukoc Biol.

[51] McCartney-Francis N, Jin W, Wahl SM (2004). Aberrant Toll receptor expression and endotoxin hypersensitivity in mice lacking a functional TGF-β1 signaling pathway. J Immunol.

[52] Henderson NC, Sheppard D (2013). Integrin-mediated regulation of TGFβ in fibrosis. Biochimi Biophys Acta.

[53] Bannerman DD, Paape MJ, Chockalingam A (2006). *Staphylococcus aureus* intramammary infection elicits increased production of transforming growth factor-a, -β1, and -β2. Vet Immunol Immunopathol.

[54] Straub RH (2006). The complex role of estrogens in inflammation. Endocr Rev.

[55] Ng THS (2013). Regulation of adaptive immunity; the role of Interleukin-10. Front Immunol.

[56] Xie L (2014). MKL1/2 and ELK4 co-regulate distinct serum response factor (SRF) transcription programs in macrophages. BMC Genomics.

[57] Liu C, Adamson E, Mercola D (1996). Transcription factor EGR-1 suppresses the growth and transformation of human HT-1080 fibrosarcoma cells by induction of transforming growth factor beta 1. Proc Natl Acad Sci USA.

[58] Hayden MS, Ghosh S (2014). Regulation of NF-κB by TNF family cytokines. Semin Immunol.

[59] Popoff MR (2014). Bacterial factors exploit eukaryotic Rho GTPase signaling cascades to promote invasion and proliferation within their host. Small GTPases.

[60] Schröder A (2006). *Staphylococcus aureus* fibronectin binding protein-a induces motile attachment sites and complex actin remodeling in living endothelial Cells. Mol Biol Cell.

[61] Sinha B (1999). Fibronectin-binding protein acts as *Staphylococcus aureus* invasin via fibronectin bridging to integrin α5β1. Cellul Microbiol.

[62] Döpfer D (2000). Adhesion and invasion of *Escherichia coli* from single and recurrent clinical cases of bovine mastitis *in vitro*. Vet Microbiol.

[63] Hensen SM, Pavicic MJ, Lohuis JA, Poutrel B (2000). Use of bovine primary mammary epithelial cells for the comparison of adherence and invasion ability of *Staphylococcus aureus* strains. J Dairy Sci.

[64] Leimbach A (2015). Complete genome sequences of *Escherichia coli* Strains 1303 and ECC-1470 isolated from bovine mastitis. Genome Announc.

[65] Wolf C (2011). Genomic and proteomic characterization of *Staphylococcus aureus* mastitis isolates of bovine origin. Proteomics.

[66] Nieto C, Espinosa M (2003). Construction of the mobilizable plasmid pMV158GFP, a derivative of pMV158 that carries the gene encoding the green fluorescent protein. Plasmid.

[67] Dogan B (2006). Adherent and invasive *Escherichia coli* are associated with persistent bovine mastitis. Vet Microbiol.

