# Supplementary material for: Differentiating Staphylococcus aureus from Escherichia coli mastitis: S. aureus triggers unbalanced immune-dampening and host cell invasion immediately after udder infection
Source: Sci Rep. 2017 Jul 6;7:4811. doi: 10.1038/s41598-017-05107-4 (PMC5500526; doi:10.1038/s41598-017-05107-4)
Supplement: Supplementary file 1 — Supplementary material [file 41598_2017_5107_MOESM1_ESM.pdf]

Supplementary Material

Differentiating *Staphylococcus aureus* from *Escherichia coli* mastitis: *S. aureus* triggers unbalanced immune-dampening and host cell invasion immediately after udder infection.

Juliane Günther, Wolfram Petzl, Isabel Bauer, Siriluck Ponsuksili, Holm Zerbe, Hans-Joachim Schuberth, Ronald M. Brunner, Hans-Martin Seyfert

*** Correspondence:** Hans-Martin Seyfert: seyfert@fbn-dummerstorf.de

Supplementary Figures

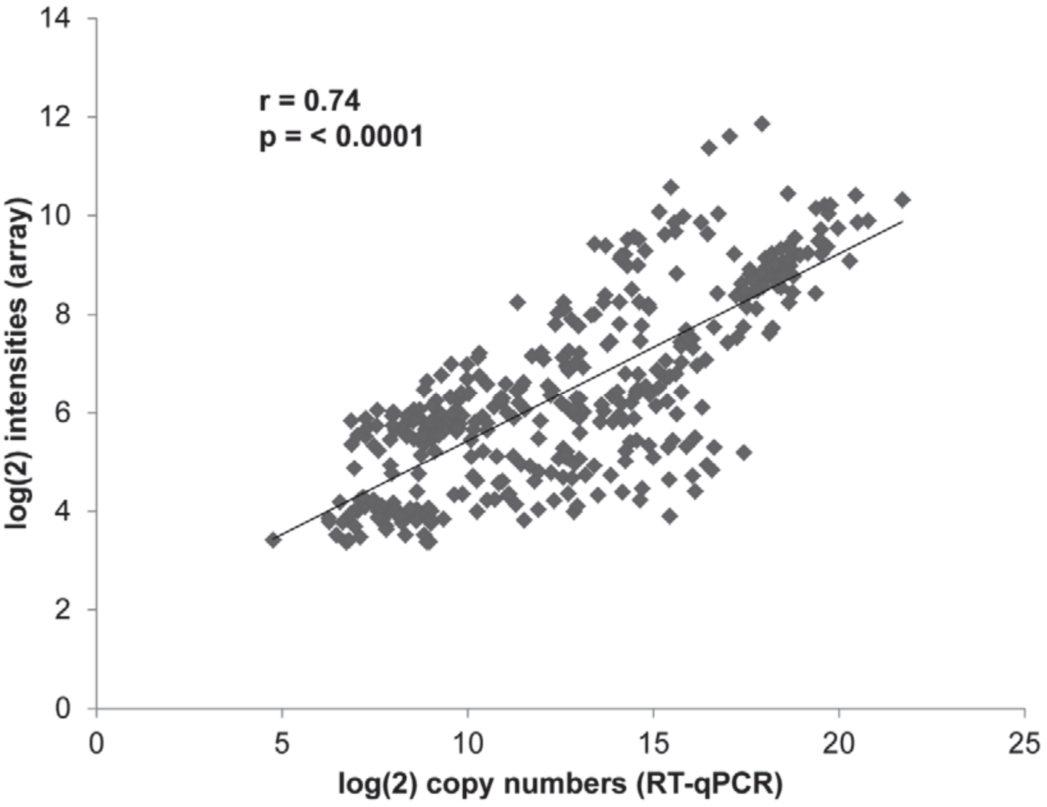

Supplementary Figure 1. Correlation of mRNA abundance determined by microarray and RT-qPCR measurements. Dot plot showing the correlations between expression levels of MX2, LCN2, CCL20, TNF, LAP, S100A9, IL6, and CXCL8 in all 48 gland cistern samples determined by microarray analysis [ordinate; log(2) intensities] or by RT-qPCR [abscissa; log(2) copy numbers]. Spearman correlation coefficient (r) and two-tailed p-value (p) are shown in the graph.

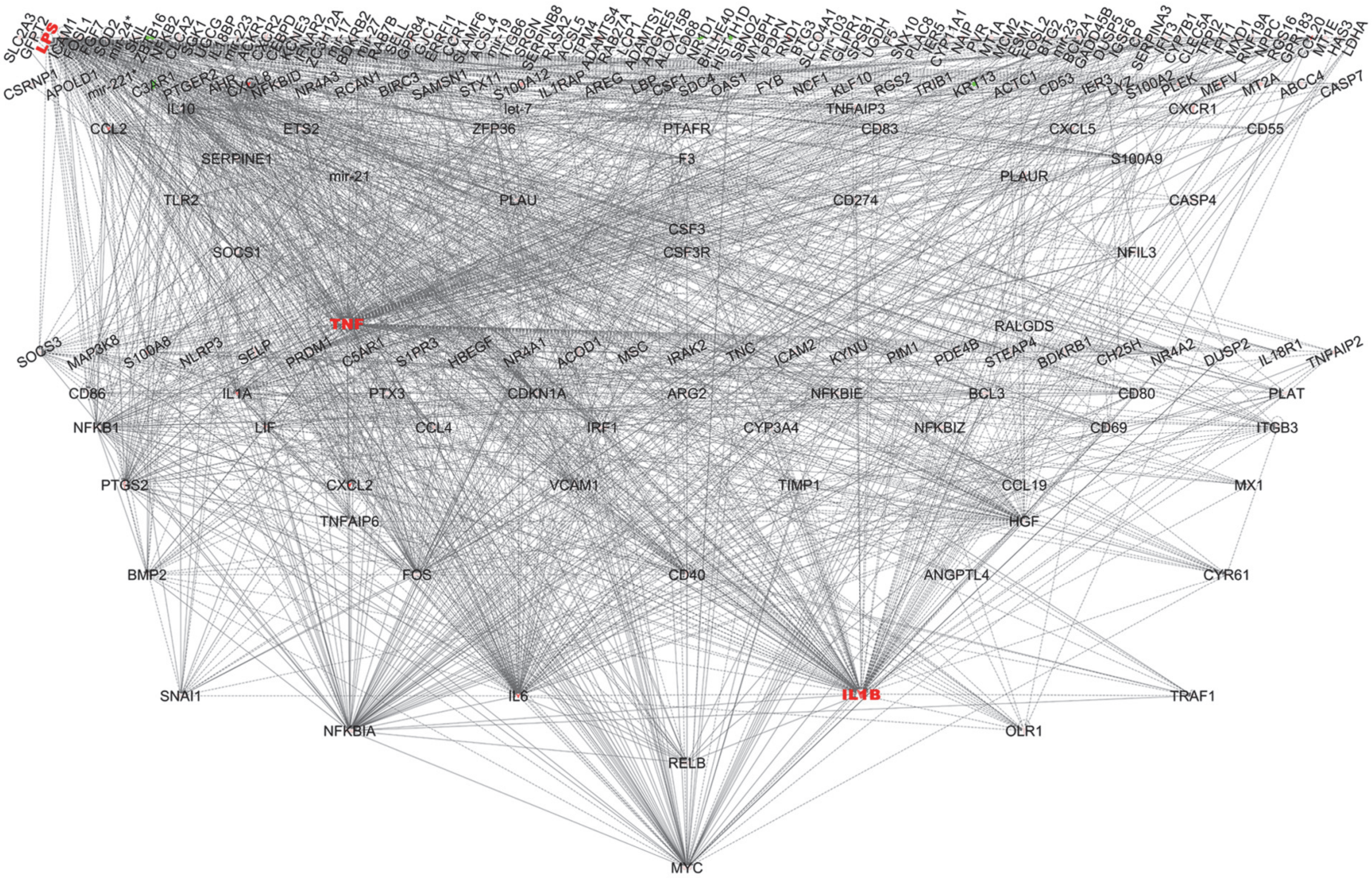

Supplementary Figure 2. Graphical display of the regulatory network governing the response towards an *E. coli* infection. Regulatory networks triggered through the three dominant upstream regulators LPS, IL1B, and TNF (red, cf. Figure 2A of the main text), as generated with the Ingenuity software.

Supplementary Tables

Supplementary Table 1. List of all DEGs, sorted according to the infecting pathogen and the duration of infection. Transcripts were defined as DEGs if their expression in the infected quarter was > 1.5 fold that of the unstimulated control quarter and the p-value of the univariate t-test paired according the individual animal was < 0.05. The false discovery rate (FDR) is also indicated.

	Probe set	Gene ID	Gene	Gene name	fold change	Parametric p-value	FDR
E. coli 1 h							
	12698536	281860	IL1RN	interleukin 1 receptor antagonist	**1.7**	0.047652	0.919
	12693115	100298391	LOC100298391	hypothetical protein LOC100298391	**-1.7**	0.014347	0.919
	12728239	518561	LOC518561	similar to olfactory receptor 561 (predicted)	**-1.7**	0.044371	0.919
E. coli 2 h							
	12848835	280826	IL6	interleukin 6 (interferon, beta 2)	**14.5**	0.000026	0.008
	12703842	281251	IL1B	interleukin 1, beta	**11.2**	0.000326	0.018
	12767023	281043	CCL2	chemokine (C-C motif) ligand 2	**11.0**	0.002887	0.051
	12836080	286806	ADAMTS4	ADAM metallopeptidase with thrombospondin type 1 motif, 4	**9.1**	0.000020	0.008
	12869838	280828	IL8	interleukin 8	**9.1**	0.000454	0.020
	12858617	508869	RND1	Rho family GTPase 1	**9.1**	0.000254	0.018
	12742653	514346	SDS	serine dehydratase	**9.1**	0.000280	0.018
	12774476	281666	CCL20	chemokine (C-C motif) ligand 20	**8.3**	0.000481	0.021
	12869915	281214	CXCL2	chemokine (C-X-C motif) ligand 2	**8.3**	0.002953	0.052
	12698536	281860	IL1RN	interleukin 1 receptor antagonist	**8.3**	0.000317	0.018
	12820239	529092	LOC529092	similar to RIKEN cDNA 9930023K05	**8.3**	0.004980	0.070
	12718324	505080	TGM3	transglutaminase 3 (E polypeptide, protein-glutamine-gamma-glutamyltransferase)	**8.3**	0.000621	0.023
	12703833	281250	IL1A	interleukin 1, alpha	**7.7**	0.002278	0.045
	12711935	517231	MGC165939	Uncharacterized protein C13orf18 homolog	**7.7**	0.000214	0.018
	12841346	616818	S100A8	S100 calcium binding protein A8	**7.7**	0.000848	0.026
	12779840	12779841	CXCR1	chemokine (C-X-C motif) receptor 1	**7.7**	0.000111	0.014
	12788935	100337435	LOC100337435	TNFAIP2 protein-like	**7.1**	0.001177	0.034
	12836069	282467	S100A12	S100 calcium binding protein A12 (calgranulin C)	**7.1**	0.000450	0.020
	12906575	100313022	MIR223	microRNA mir-223	**5.9**	0.000568	0.022
	12876963	281839	ICAM1	intercellular adhesion molecule 1	**5.6**	0.000085	0.014
	12738576	282023	PTGS2	prostaglandin-endoperoxide synthase 2 (prostaglandin G/H synthase and cyclooxygenase)	**5.6**	0.002663	0.050
	12832640	281474	SAA3	serum amyloid A 3	**5.6**	0.002336	0.046
	12784351	616377	CDA	cytidine deaminase	**5.3**	0.000125	0.014
	12869909	281212	CXCL2	chemokine (C-X-C motif) ligand 2	**5.3**	0.002972	0.052
	12817572	529195	MEFV	Mediterranean fever	**5.3**	0.004490	0.066
	12739133	508958	SLC26A9	solute carrier family 26, member 9	**5.3**	0.000356	0.018
	12880314	530101	GFPT2	glutamine-fructose-6-phosphate transaminase 2	**5.0**	0.000694	0.024
	12861876	540044	GPR84	G protein-coupled receptor 84	**5.0**	0.001733	0.040

	12766656	100140873	ICAM2	intercellular adhesion molecule 2	**5.0**	0.000126	0.014
	12756033	404070	MT2A	metallothionein 2A	**5.0**	0.000796	0.025
	12788161	539780	NAIP	NLR family, apoptosis inhibitory protein	**5.0**	0.000447	0.020
	12738559	281486	SELP	selectin P	**5.0**	0.000289	0.018
	12901691	534871	TNFAIP2	tumor necrosis factor, alpha-induced protein 2	**5.0**	0.000753	0.025
	12877288	337917	IRF1	interferon regulatory factor 1	**4.8**	0.000334	0.018
	12755776	281983	PLAUR	plasminogen activator, urokinase receptor	**4.8**	0.000805	0.025
	12767029	281044	CCL8	chemokine (C-C motif) ligand 8	**4.5**	0.009260	0.106
	12870010	281735	CXCL5	chemokine (C-X-C motif) ligand 5	**4.5**	0.000240	0.018
	12875339	538691	HS3ST1	heparan sulfate (glucosamine) 3-O-sulfotransferase 1	**4.5**	0.000340	0.018
	12745282	280840	LIF	leukemia inhibitory factor (cholinergic differentiation factor)	**4.5**	0.001360	0.036
	12812172	510394	PRSS22	protease, serine, 22	**4.5**	0.000362	0.018
	12900969	534929	VNN2	vanin 2	**4.5**	0.001113	0.033
	12713828	286849	CD40	CD40 molecule, TNF receptor superfamily member 5	**4.3**	0.000096	0.014
	12837529	509860	S100A2	S100 calcium binding protein A2	**4.3**	0.001686	0.040
	12735710	516303	ERRFI1	ERBB receptor feedback inhibitor 1	**4.2**	0.000270	0.018
	12700364	518658	PLEK	pleckstrin	**4.2**	0.001178	0.034
	12805551	514076	CFB	complement factor B	**4.0**	0.000324	0.018
	12862854	781146	LOC781146	lysozyme	**4.0**	0.001440	0.037
	12752709	531942	BCL3	B-cell CLL/lymphoma 3	**3.8**	0.000001	0.003
	12806784	617034	CD83	CD83 molecule	**3.8**	0.001945	0.043
	12837790	511511	CSF3R	similar to colony stimulating factor 3 receptor	**3.8**	0.000530	0.022
	12690238	529849	FAM148A	C2 calcium-dependent domain containing 4B	**3.8**	0.000317	0.018
	12755625	280692	HP	haptoglobin	**3.8**	0.000740	0.025
	12779639	782719	IL8RB	interleukin 8 receptor, beta	**3.8**	0.004031	0.062
	12845099	518597	LOC518597	similar to Uncharacterized protein C1orf161 homolog	**3.8**	0.002808	0.051
	12846075	532569	S100A9	S100 calcium binding protein A9	**3.8**	0.000258	0.018
	12896970	508105	TNFAIP3	tumor necrosis factor, alpha-induced protein 3	**3.7**	0.001138	0.034
	12709146	504806	IRG1	immunoresponsive 1 homolog (mouse)	**3.6**	0.000687	0.024
	12842360	100296588	LOC100296588	hypothetical protein LOC100296588	**3.4**	0.001562	0.038
	12804428	280943	TNF	tumor necrosis factor (TNF superfamily, member 2)	**3.4**	0.035854	0.253
	12767838	414347	CCL4	chemokine (C-C motif) ligand 4	**3.3**	0.002859	0.051
	12707400	539140	DUSP2	dual specificity phosphatase 2	**3.3**	0.000021	0.008
	12751936	517354	LOC517354	similar to fractalkine	**3.3**	0.001501	0.037
	12760916	282081	SOCS3	suppressor of cytokine signaling 3	**3.3**	0.000125	0.014
	12885365	514246	JUNB	jun B proto-oncogene	**3.2**	0.000740	0.025
	12716461	535622	MAP3K8	mitogen-activated protein kinase kinase kinase 8	**3.2**	0.000242	0.018
	12826119	509501	SRGN	serglycin	**3.2**	0.002177	0.044
	12856817	281058	CD69	CD69 molecule	**3.1**	0.002566	0.049
	12730037	617470	IL18BP	interleukin 18 binding protein	**3.0**	0.000473	0.021
	12780275	281863	IL8RB	interleukin 8 receptor, beta	**3.0**	0.002201	0.044
	12869555	100295476	LOC100295476	similar to epiregulin preproprotein	**3.0**	0.002342	0.046
	12868930	777776	LYZ	lysozyme (renal amyloidosis)	**3.0**	0.005956	0.081
	12718118	282431	PROKR2	prokineticin receptor 2	**3.0**	0.011770	0.125

	12718754	507969	SLCO4A1	solute carrier organic anion transporter family, member 4A1	**3.0**	0.001814	0.041
	12681452	534171	SAMSN1	SAM domain, SH3 domain and nuclear localization signals 1	**2.9**	0.003244	0.053
	12801900	513497	CDKN1A	cyclin-dependent kinase inhibitor 1A (p21, Cip1)	**2.9**	0.000025	0.008
	12758131	526736	KLK10	kallikrein-related peptidase 10	**2.9**	0.000012	0.008
	12791746	282291	NFKBIA	nuclear factor of kappa light polypeptide gene enhancer in B-cells inhibitor, alpha	**2.9**	0.001286	0.035
	12815205	281375	SERPINE1	serpin peptidase inhibitor, clade E (nexin, plasminogen activator inhibitor type 1), member 1	**2.9**	0.003718	0.059
	12878460	509963	ANGPTL4	angiopoietin-like 4	**2.8**	0.000290	0.018
	12791724	282151	BCL2A1	BCL2-related protein A1	**2.8**	0.000249	0.018
	12732071	514386	BIRC3	baculoviral IAP repeat-containing 3	**2.8**	0.002740	0.050
	12894605	533834	CD274	CD274 molecule	**2.8**	0.001672	0.040
	12743274	527744	LOC527744	similar to G protein-coupled receptor 109B	**2.8**	0.001573	0.038
	12756039	404071	MT1A	metallothionein 1E	**2.8**	0.000570	0.022
	12686574	541148	PTX3	pentraxin-related gene, rapidly induced by IL-1 beta	**2.8**	0.003037	0.053
	12738588	282035	RGS16	regulator of G-protein signaling 16	**2.8**	0.002855	0.051
	12907975	511661	SLC6A14	solute carrier family 6 (amino acid transporter), member 14	**2.8**	0.000744	0.025
	12898561	539960	STX11	syntaxin 11	**2.7**	0.002048	0.044
	12819271	507061	DUSP5	similar to dual specificity phosphatase 5	**2.6**	0.000136	0.014
	12786321	539571	ESM1	endothelial cell-specific molecule 1	**2.6**	0.010629	0.116
	12749590	286824	HAS3	hyaluronan synthase 3	**2.6**	0.001271	0.035
	12901685	511695	LOC511695	similar to serpin peptidase inhibitor, clade B like	**2.6**	0.019674	0.168
	12749424	282127	ZFP36	zinc finger protein 36, C3H type, homolog (mouse)	**2.6**	0.000371	0.018
	12791970	507093	ALDH1A3	aldehyde dehydrogenase 1 family, member A3	**2.6**	0.004191	0.065
	12794515	407771	CCR1	chemokine (C-C motif) receptor 1	**2.6**	0.007976	0.095
	12835715	280686	F3	coagulation factor III (thromboplastin, tissue factor)	**2.6**	0.002093	0.044
	12834336	531389	FOSL1	FOS-like antigen 1	**2.6**	0.001403	0.037
	12780177	281356	NPPC	natriuretic peptide precursor C	**2.6**	0.025218	0.194
	12911085	31340899	SERPINA3-1	Bos taurus serpin peptidase inhibitor, clade A (alpha-1 antiproteinase, antitrypsin), member 3 (SERPINA3-1), mRNA.	**2.6**	0.005698	0.079
	12760699	281096	CSF3	colony stimulating factor 3 (granulocyte)	**2.5**	0.000094	0.014
	12825057	510037	RNF122	ring finger protein 122	**2.5**	0.000104	0.014
	12893422	511556	SLC46A2	solute carrier family 46, member 2	**2.5**	0.019005	0.165
	12714026	504492	SNAI1	snail homolog 1 (Drosophila)	**2.5**	0.000788	0.025
	12897137	512044	ACAT2	acetyl-Coenzyme A acetyltransferase 2	**2.4**	0.002335	0.046
	12721602	281678	CEBPD	CCAAT/enhancer binding protein (CEBP), delta	**2.4**	0.000117	0.014
	12870009	281212	CXCL2	chemokine (C-X-C motif) ligand 2	**2.4**	0.001723	0.040
	12807296	100296226	LOC100296226	similar to phorbol-12-myristate-13-acetate-induced protein 1	**2.4**	0.000909	0.027
	12875758	616115	NFKB1	nuclear factor of kappa light polypeptide gene enhancer in B-cells 1	**2.4**	0.000546	0.022
	12856897	281368	OLR1	oxidized low density lipoprotein (lectin-like) receptor 1	**2.4**	0.000190	0.017
	12738550	281485	SELL	selectin L	**2.4**	0.001221	0.035
	12846451	535344	ZC3H12A	zinc finger CCCH-type containing 12A	**2.4**	0.002550	0.049
	12693192	100313003	MIR147	microRNA mir-147	**2.4**	0.021404	0.176
	12824753	281407	PLAT	plasminogen activator, tissue	**2.4**	0.000228	0.018
	12733548	616239	TCN1	transcobalamin I (vitamin B12 binding protein, R binder family)	**2.4**	0.001382	0.036
	12724848	511077	MYC	v-myc myelocytomatosis viral oncogene homolog (avian)	**2.3**	0.000039	0.011
	12788637	100272170	SERPINA3-2	SERPIN A3-2	**2.3**	0.008389	0.098

	12760699	281096	CSF3	colony stimulating factor 3 (granulocyte)	**2.5**	0.000094	0.014
	12825057	510037	RNF122	ring finger protein 122	**2.5**	0.000104	0.014
	12893422	511556	SLC46A2	solute carrier family 46, member 2	**2.5**	0.019005	0.165
	12714026	504492	SNAI1	snail homolog 1 (Drosophila)	**2.5**	0.000788	0.025
	12897137	512044	ACAT2	acetyl-Coenzyme A acetyltransferase 2	**2.4**	0.002335	0.046
	12721602	281678	CEBPD	CCAAT/enhancer binding protein (CEBP), delta	**2.4**	0.000117	0.014
	12870009	281212	CXCL2	chemokine (C-X-C motif) ligand 2	**2.4**	0.001723	0.040
	12807296	100296226	LOC100296226	similar to phorbol-12-myristate-13-acetate-induced protein 1	**2.4**	0.000909	0.027
	12875758	616115	NFKB1	nuclear factor of kappa light polypeptide gene enhancer in B-cells 1	**2.4**	0.000546	0.022
	12856897	281368	OLR1	oxidized low density lipoprotein (lectin-like) receptor 1	**2.4**	0.000190	0.017
	12738550	281485	SELL	selectin L	**2.4**	0.001221	0.035
	12846451	535344	ZC3H12A	zinc finger CCCH-type containing 12A	**2.4**	0.002550	0.049
	12693192	100313003	MIR147	microRNA mir-147	**2.4**	0.021404	0.176
	12824753	281407	PLAT	plasminogen activator, tissue	**2.4**	0.000228	0.018
	12733548	616239	TCN1	transcobalamin I (vitamin B12 binding protein, R binder family)	**2.4**	0.001382	0.036
	12724848	511077	MYC	v-myc myelocytomatosis viral oncogene homolog (avian)	**2.3**	0.000039	0.011
	12788637	100272170	SERPINA3-2	SERPIN A3-2	**2.3**	0.008389	0.098
	12864151	282358	SLC2A3	solute carrier family 2 (facilitated glucose transporter), member 3	**2.3**	0.000727	0.025
	12842594	281094	CSF1	colony stimulating factor 1 (macrophage)	**2.2**	0.002170	0.044
	12762310	508666	LOC508666	similar to C-C motif chemokine 23 precursor	**2.2**	0.006271	0.084
	12736300	523618	IER5	immediate early response 5	**2.2**	0.002178	0.044
	12906283	790984	MIR222	microRNA mir-222	**2.2**	0.001943	0.043
	12906581	100313062	MIR505	microRNA mir-505	**2.2**	0.012716	0.130
	12878613	510803	PGLYRP2	peptidoglycan recognition protein 2	**2.2**	0.010579	0.116
	12775981	509774	RNF19B	ring finger protein 19B	**2.2**	0.000024	0.008
	12718768	508133	SDC4	syndecan 4	**2.2**	0.003996	0.062
	12798575	506945	BHLHB2	basic helix-loop-helix domain containing, class B, 2	**2.1**	0.005393	0.075
	12803236	539635	FAM65B	family with sequence similarity 65, member B	**2.1**	0.001508	0.037
	12705036	509889	FOSL2	FOS-like antigen 2	**2.1**	0.000087	0.014
	12801358	505455	IER3	immediate early response 3	**2.1**	0.011573	0.124
	12825859	281408	PLAU	plasminogen activator, urokinase	**2.1**	0.001962	0.043
	12726914	404129	DGAT2	diacylglycerol O-acyltransferase homolog 2 (mouse)	**2.1**	0.006665	0.087
	12885817	522921	HBEGF	heparin-binding EGF-like growth factor	**2.1**	0.000157	0.015
	12814908	100140740	IGSF6	immunoglobulin superfamily, member 6	**2.1**	0.003262	0.053
	12766478	790982	MIR21	microRNA mir-21	**2.1**	0.023696	0.187
	12752266	522670	RELB	v-rel reticuloendotheliosis viral oncogene homolog B	**2.1**	0.000222	0.018
	12855397	538861	STEAP4	STEAP family member 4	**2.1**	0.006825	0.087
	12761264	286820	ALOX15B	arachidonate 15-lipoxygenase, type B	**2.0**	0.028195	0.212
	12768239	506088	ICAM2	intercellular adhesion molecule 2	**2.0**	0.018077	0.160
	12705580	513723	RALGDS	ral guanine nucleotide dissociation stimulator	**2.0**	0.000375	0.018
	12725595	529552	CYP7B1	cytochrome P450, family 7, subfamily B, polypeptide 1	**2.0**	0.003125	0.053
	12818707	100139670	IFIT1	interferon-induced protein with tetratricopeptide repeats 1	**2.0**	0.024074	0.188
	12889469	506097	NFIL3	nuclear factor, interleukin 3 regulated	**2.0**	0.004459	0.066
	12845740	528076	DARC	Duffy blood group, chemokine receptor	**2.0**	0.001724	0.040

	12906281	790983	MIR221	microRNA mir-221	**2.0**	0.018312	0.160
	12901108	538384	PRDM1	PR domain containing 1, with ZNF domain	**2.0**	0.000132	0.014
	12822921	538469	SLC16A12	solute carrier family 16, member 12 (monocarboxylic acid transporter 12)	**2.0**	0.016476	0.150
	12780587	493710	TNFAIP6	tumor necrosis factor, alpha-induced protein 6	**2.0**	0.017873	0.159
	12796519	537051	ADAMTS9	ADAM metallopeptidase with thrombospondin type 1 motif, 9	**1.9**	0.005963	0.081
	12682289	613977	LOC613977	similar to TPA-induced transmembrane protein	**1.9**	0.002617	0.049
	12890889	528877	NR4A3	nuclear receptor subfamily 4, group A, member 3	**1.9**	0.022222	0.180
	12682704	782774	P2RY13	purinergic receptor P2Y, G-protein coupled, 13	**1.9**	0.024672	0.192
	12740535	538050	RAB7B	RAB7B, member RAS oncogene family	**1.9**	0.002727	0.050
	12899456	281496	SOD2	superoxide dismutase 2, mitochondrial	**1.9**	0.003732	0.059
	12745324	281534	TLR2	toll-like receptor 2	**1.9**	0.000235	0.018
	12864466	504244	TUBA1C	tubulin, alpha 1c	**1.9**	0.003243	0.053
	12820145	526392	NFKB2	nuclear factor of kappa light polypeptide gene enhancer in B-cells 2 (p49/p100)	**1.9**	0.000005	0.005
	12739170	509732	PDPN	podoplanin	**1.9**	0.039879	0.273
	12819421	509678	IFIT3	interferon-induced protein with tetratricopeptide repeats 3	**1.9**	0.011060	0.119
	12683452	282258	IFNAR2	interferon (alpha, beta and omega) receptor 2	**1.9**	0.004742	0.068
	12915093	297468078	LOC786591	PREDICTED: Bos taurus Rho-type GTPase-activating protein FLJ32810 (LOC786591), partial mRNA.	**1.9**	0.030088	0.224
	12881023	538639	NLRP3	NLR family, pyrin domain containing 3	**1.9**	0.020409	0.172
	12681965	539640	RCAN1	regulator of calcineurin 1	**1.9**	0.002083	0.044
	12739756	518609	CD55	CD55 molecule, decay accelerating factor for complement (Cromer blood group)	**1.8**	0.018552	0.162
	12749099	100336600	LOC100336600	solute carrier family 7 (cationic amino acid transporter, y+ system), member 3-like	**1.8**	0.007299	0.092
	12837291	508183	SLAMF6	SLAM family member 6	**1.8**	0.021904	0.178
	12725414	521857	TRIB1	tribbles homolog 1 (Drosophila)	**1.8**	0.006624	0.087
	12820137	526279	CASP7	caspase 7, apoptosis-related cysteine peptidase	**1.8**	0.000759	0.025
	12700012	514626	MXD1	MAX dimerization protein 1	**1.8**	0.002758	0.050
	12790904	538896	BDKRB2	bradykinin receptor B2	**1.7**	0.001838	0.041
	12678583	281148	ETS2	v-ets erythroblastosis virus E26 oncogene homolog 2 (avian)	**1.7**	0.000522	0.022
	12759042	613358	MT1E	metallothionein 1E	**1.7**	0.002085	0.044
	12875956	767910	PLAC8	placenta-specific 8	**1.7**	0.000083	0.014
	12696433	538143	RHOV	ras homolog gene family, member V	**1.7**	0.024730	0.192
	12737028	539364	BTG2	BTG family, member 2	**1.7**	0.000559	0.022
	12687797	100313119	MIR2292	microRNA mir-2292	**1.7**	0.003544	0.057
	12678828	282713	NFKBIZ	nuclear factor of kappa light polypeptide gene enhancer in B-cells inhibitor, zeta	**1.7**	0.031396	0.229
	12900295	515854	SGK1	serum/glucocorticoid regulated kinase 1	**1.7**	0.020677	0.173
	12832069	767970	ASRGL1	asparaginase like 1	**1.7**	0.006351	0.085
	12682216	541054	BTG3	BTG family, member 3	**1.7**	0.005338	0.075
	12799096	515406	CSRNP1	cysteine-serine-rich nuclear protein 1	**1.7**	0.000081	0.014
	12805054	506727	NFKBIE	nuclear factor of kappa light polypeptide gene enhancer in B-cells inhibitor, epsilon	**1.7**	0.000432	0.020
	12842051	100124505	PDE4B	phosphodiesterase 4B, cAMP-specific (phosphodiesterase E4 dunce homolog, Drosophila)	**1.7**	0.011667	0.124
	12738745	504407	SPSB1	splA/ryanodine receptor domain and SOCS box containing 1	**1.7**	0.006535	0.086
	12689697	518752	ARG2	arginase, type II	**1.6**	0.014887	0.145
	12683618	407131	CD80	CD80 molecule	**1.6**	0.000090	0.014
	12786143	535347	FYB	FYN binding protein (FYB-120/130)	**1.6**	0.037163	0.259
	12888026	618405	GADD45B	growth arrest and DNA-damage-inducible, beta	**1.6**	0.009679	0.109

	12714733	512242	LBP	lipopolysaccharide binding protein	**1.6**	0.007551	0.094
	12749684	407171	LOC407171	Fc gamma 2 receptor	**1.6**	0.003796	0.060
	12752468	527020	LOC527020	similar to Immunoglobulin superfamily member 1 precursor	**1.6**	0.024098	0.188
	12879457	520348	MARCH3	membrane-associated ring finger (C3HC4) 3	**1.6**	0.006748	0.087
	12680419	514613	MCM2	cyclin L1	**1.6**	0.003086	0.053
	12752457	526865	PVR	poliovirus receptor	**1.6**	0.006830	0.087
	12791243	617667	SERPINA3	serpin peptidase inhibitor, clade A (alpha-1 antiproteinase, antitrypsin), member 3	**1.6**	0.009694	0.109
	12716976	615037	BMP2	bone morphogenetic protein 2	**1.6**	0.001458	0.037
	12799059	515034	IRAK2	interleukin-1 receptor-associated kinase 2	**1.6**	0.001486	0.037
	12877819	505903	LOC505903	similar to histone H2B	**1.6**	0.018205	0.160
	12915197	297468753	LOC783052	PREDICTED: Bos taurus elastin microfibril interfacer 2-like (LOC783052), partial mRNA.	**1.6**	0.005391	0.075
	12876519	100313233	MIR2462	microRNA mir-2462	**1.6**	0.007811	0.094
	12804472	281402	PIM1	pim-1 oncogene	**1.6**	0.008049	0.095
	12769871	512652	SSH2	slingshot homolog 2 (Drosophila)	**1.6**	0.009030	0.104
	12895180	540944	TRAF1	TNF receptor-associated factor 1	**1.6**	0.002160	0.044
	12915081	297467992	LOC100294770	PREDICTED: Bos taurus hypothetical LOC100294770 (LOC100294770), mRNA.	**1.6**	0.007825	0.094
	12915137	297468411	LOC100335378	PREDICTED: Bos taurus hypothetical protein LOC100335378 (LOC100335378), mRNA.	**1.6**	0.007825	0.094
	12915359	297469511	LOC100335892	PREDICTED: Bos taurus hypothetical protein LOC100335892 (LOC100335892), mRNA.	**1.6**	0.007825	0.094
	12817048	518795	SOCS1	suppressor of cytokine signaling 1	**1.6**	0.003262	0.053
	12875039	533681	TMEM156	transmembrane protein 156	**1.6**	0.000433	0.020
	12843460	506550	TSPAN1	tetraspanin 1	**1.6**	0.044203	0.291
	12893218	509167	CCL19	chemokine (C-C motif) ligand 19	**1.6**	0.000144	0.015
	12843223	505040	CD53	CD53 molecule	**1.6**	0.006035	0.082
	12855777	615201	CLEC5A	C-type lectin domain family 5, member A	**1.6**	0.000318	0.018
	12731455	508009	FLJ32810	hypothetical protein LOC508009	**1.6**	0.015052	0.145
	12711686	510187	MGC155285	Uncharacterized protein C13orf33 homolog	**1.6**	0.040734	0.276
	12692187	618035	RAB27A	RAB27A, member RAS oncogene family	**1.6**	0.006291	0.084
	12722742	522795	KLF10	Kruppel-like factor 10	**1.5**	0.039674	0.273
	12841505	618250	S100A14	S100 calcium binding protein A14	**1.5**	0.023280	0.185
	12867769	538827	APOLD1	apolipoprotein L domain containing 1	**1.5**	0.007407	0.093
	12788556	784765	GAPT	GRB2-binding adaptor protein, transmembrane	**1.5**	0.018148	0.160
	12774621	282644	ITGB6	integrin, beta 6	**1.5**	0.035978	0.253
	12788794	100313057	MIR493	microRNA mir-493	**1.5**	0.013040	0.132
	12759088	614198	NFKBID	nuclear factor of kappa light polypeptide gene enhancer in B-cells inhibitor, delta	**1.5**	0.000003	0.003
	12896203	100139174	POPDC3	popeye domain containing 3	**-1.6**	0.047851	0.304
	12765513	616622	PRR15L	proline rich 15-like	**-1.6**	0.003199	0.053
	12868116	540702	C3AR1	complement component 3a receptor 1	**-1.6**	0.004792	0.068
	12896448	100336854	LOC100336854	hypothetical protein LOC100336854	**-1.7**	0.015701	0.145
	12705776	515213	CLIP4	CAP-GLY domain containing linker protein family, member 4	**-1.7**	0.029013	0.217
***E. coli* 3 h**							
	12869915	281214	CXCL2	chemokine (C-X-C motif) ligand 2	**21.3**	0.002905	0.151
	12774476	281666	CCL20	chemokine (C-C motif) ligand 20	**18.2**	0.001719	0.151
	12869838	280828	IL8	interleukin 8	**17.9**	0.000775	0.151

	12848835	280826	IL6	interleukin 6 (interferon, beta 2)	**15.4**	0.007411	0.151
	12703842	281251	IL1B	interleukin 1, beta	**13.9**	0.002015	0.151
	12767023	281043	CCL2	chemokine (C-C motif) ligand 2	**13.7**	0.001204	0.151
	12858617	508869	RND1	Rho family GTPase 1	**12.3**	0.000848	0.151
	12703833	281250	IL1A	interleukin 1, alpha	**12.0**	0.005014	0.151
	12869909	281212	CXCL2	chemokine (C-X-C motif) ligand 2	**11.9**	0.002526	0.151
	12698536	281860	IL1RN	interleukin 1 receptor antagonist	**10.3**	0.004345	0.151
	12820239	529092	LOC529092	similar to RIKEN cDNA 9930023K05	**10.0**	0.006019	0.151
	12745282	280840	LIF	leukemia inhibitory factor (cholinergic differentiation factor)	**9.1**	0.002172	0.151
	12832640	281474	SAA3	serum amyloid A 3	**9.1**	0.002418	0.151
	12738576	282023	PTGS2	prostaglandin-endoperoxide synthase 2 (prostaglandin G/H synthase and cyclooxygenase)	**8.3**	0.008818	0.151
	12718324	505080	TGM3	transglutaminase 3 (E polypeptide, protein-glutamine-gamma-glutamyltransferase)	**8.3**	0.029879	0.194
	12804428	280943	TNF	tumor necrosis factor (TNF superfamily, member 2)	**8.3**	0.020693	0.185
	12836080	286806	ADAMTS4	ADAM metallopeptidase with thrombospondin type 1 motif, 4	**7.7**	0.007322	0.151
	12861876	540044	GPR84	G protein-coupled receptor 84	**7.7**	0.016841	0.175
	12711935	517231	MGC165939	Uncharacterized protein C13orf18 homolog	**7.7**	0.021911	0.186
	12841346	616818	S100A8	S100 calcium binding protein A8	**7.7**	0.009664	0.151
	12869555	100295476	LOC100295476	similar to epiregulin preproprotein	**7.1**	0.002889	0.151
	12817572	529195	MEFV	Mediterranean fever	**7.1**	0.032657	0.203
	12836069	282467	S100A12	S100 calcium binding protein A12 (calgranulin C)	**7.1**	0.012096	0.159
	12742653	514346	SDS	serine dehydratase	**7.1**	0.018077	0.178
	12788935	100337435	LOC100337435	TNFAIP2 protein-like	**6.7**	0.005362	0.151
	12779840	12779841	CXCR1	chemokine (C-X-C motif) receptor 1	**6.7**	0.029641	0.194
	12767029	281044	CCL8	chemokine (C-C motif) ligand 8	**6.3**	0.002707	0.151
	12875339	538691	HS3ST1	heparan sulfate (glucosamine) 3-O-sulfotransferase 1	**6.3**	0.003523	0.151
	12755776	281983	PLAUR	plasminogen activator, urokinase receptor	**5.9**	0.008819	0.151
	12870010	281735	CXCL5	chemokine (C-X-C motif) ligand 5	**5.6**	0.002874	0.151
	12700364	518658	PLEK	pleckstrin	**5.6**	0.007082	0.151
	12805551	514076	CFB	complement factor B	**5.3**	0.001336	0.151
	12735710	516303	ERRFI1	ERBB receptor feedback inhibitor 1	**5.3**	0.001916	0.151
	12755625	280692	HP	haptoglobin	**5.3**	0.000920	0.151
	12788161	539780	NAIP	NLR family, apoptosis inhibitory protein	**5.3**	0.010944	0.155
	12767838	414347	CCL4	chemokine (C-C motif) ligand 4	**5.0**	0.006105	0.151
	12713828	286849	CD40	CD40 molecule, TNF receptor superfamily member 5	**5.0**	0.001702	0.151
	12784351	616377	CDA	cytidine deaminase	**5.0**	0.006535	0.151
	12756033	404070	MT2A	metallothionein 2A	**5.0**	0.002946	0.151
	12896970	508105	TNFAIP3	tumor necrosis factor, alpha-induced protein 3	**5.0**	0.003263	0.151
	12876963	281839	ICAM1	intercellular adhesion molecule 1	**4.8**	0.001069	0.151
	12801358	505455	IER3	immediate early response 3	**4.8**	0.004176	0.151
	12906575	100313022	MIR223	microRNA mir-223	**4.8**	0.038845	0.219
	12806784	617034	CD83	CD83 molecule	**4.5**	0.004625	0.151
	12786321	539571	ESM1	endothelial cell-specific molecule 1	**4.5**	0.007586	0.151
	12880314	530101	GFPT2	glutamine-fructose-6-phosphate transaminase 2	**4.5**	0.020936	0.185
	12842360	100296588	LOC100296588	hypothetical protein LOC100296588	**4.5**	0.048412	0.240

	12845099	518597	LOC518597	similar to Uncharacterized protein C1orf161 homolog	**4.5**	0.022432	0.188
	12901691	534871	TNFAIP2	tumor necrosis factor, alpha-induced protein 2	**4.5**	0.007432	0.151
	12709146	504806	IRG1	immunoresponsive 1 homolog (mouse)	**4.3**	0.009059	0.151
	12837529	509860	S100A2	S100 calcium binding protein A2	**4.3**	0.010978	0.155
	12738559	281486	SELP	selectin P	**4.2**	0.020174	0.184
	12791724	282151	BCL2A1	BCL2-related protein A1	**4.0**	0.004107	0.151
	12870009	281212	CXCL2	chemokine (C-X-C motif) ligand 2	**4.0**	0.003427	0.151
	12780275	281863	IL8RB	interleukin 8 receptor, beta	**4.0**	0.039387	0.219
	12885365	514246	JUNB	jun B proto-oncogene	**4.0**	0.001177	0.151
	12751936	517354	LOC517354	similar to fractalkine	**4.0**	0.007530	0.151
	12743274	527744	LOC527744	similar to G protein-coupled receptor 109B	**4.0**	0.028835	0.194
	12815205	281375	SERPINE1	serpin peptidase inhibitor, clade E (nexin, plasminogen activator inhibitor type 1), member 1	**4.0**	0.003136	0.151
	12900969	534929	VNN2	vanin 2	**4.0**	0.048577	0.240
	12856817	281058	CD69	CD69 molecule	**3.8**	0.027115	0.193
	12766656	100140873	ICAM2	intercellular adhesion molecule 2	**3.7**	0.002921	0.151
	12877288	337917	IRF1	interferon regulatory factor 1	**3.7**	0.001597	0.151
	12812172	510394	PRSS22	protease, serine, 22	**3.7**	0.002933	0.151
	12681452	534171	SAMSN1	SAM domain, SH3 domain and nuclear localization signals 1	**3.7**	0.044086	0.231
	12911085	31340899	SERPINA3-1	Bos taurus serpin peptidase inhibitor, clade A (alpha-1 antiproteinase, antitrypsin), member 3 (SERPINA3-1), mRNA.	**3.7**	0.035708	0.211
	12707400	539140	DUSP2	dual specificity phosphatase 2	**3.6**	0.021990	0.186
	12716461	535622	MAP3K8	mitogen-activated protein kinase kinase kinase 8	**3.6**	0.001395	0.151
	12791746	282291	NFKBIA	nuclear factor of kappa light polypeptide gene enhancer in B-cells inhibitor, alpha	**3.6**	0.006575	0.151
	12846451	535344	ZC3H12A	zinc finger CCCH-type containing 12A	**3.6**	0.009510	0.151
	12779639	782719	IL8RB	interleukin 8 receptor, beta	**3.4**	0.027672	0.193
	12766478	790982	MIR21	microRNA mir-21	**3.4**	0.009757	0.151
	12791970	507093	ALDH1A3	aldehyde dehydrogenase 1 family, member A3	**3.3**	0.002102	0.151
	12732071	514386	BIRC3	baculoviral IAP repeat-containing 3	**3.3**	0.004095	0.151
	12801900	513497	CDKN1A	cyclin-dependent kinase inhibitor 1A (p21, Cip1)	**3.3**	0.011310	0.155
	12760699	281096	CSF3	colony stimulating factor 3 (granulocyte)	**3.3**	0.030024	0.194
	12856897	281368	OLR1	oxidized low density lipoprotein (lectin-like) receptor 1	**3.3**	0.013413	0.165
	12738588	282035	RGS16	regulator of G-protein signaling 16	**3.3**	0.007367	0.151
	12846075	532569	S100A9	S100 calcium binding protein A9	**3.3**	0.003085	0.151
	12739133	508958	SLC26A9	solute carrier family 26, member 9	**3.3**	0.015068	0.170
	12893422	511556	SLC46A2	solute carrier family 46, member 2	**3.3**	0.016555	0.174
	12907975	511661	SLC6A14	solute carrier family 6 (amino acid transporter), member 14	**3.3**	0.004811	0.151
	12826119	509501	SRGN	serglycin	**3.3**	0.015489	0.172
	12897137	512044	ACAT2	acetyl-Coenzyme A acetyltransferase 2	**3.2**	0.009004	0.151
	12752709	531942	BCL3	B-cell CLL/lymphoma 3	**3.2**	0.005387	0.151
	12894605	533834	CD274	CD274 molecule	**3.2**	0.023510	0.192
	12690238	529849	FAM148A	C2 calcium-dependent domain containing 4B	**3.2**	0.003947	0.151
	12907448	505052	LOC505052	similar to odorant binding protein	**3.2**	0.026980	0.193
	12765895	783343	LOC783343	similar to ULBP27	**3.2**	0.036272	0.213
	12693192	100313003	MIR147	microRNA mir-147	**3.2**	0.006532	0.151
	12825859	281408	PLAU	plasminogen activator, urokinase	**3.2**	0.012462	0.160

	12823365	100294770	LOC100294770	hypothetical protein LOC100294770	**3.1**	0.037956	0.218
	12868930	777776	LYZ	lysozyme (renal amyloidosis)	**3.1**	0.029580	0.194
	12881023	538639	NLRP3	NLR family, pyrin domain containing 3	**3.1**	0.008047	0.151
	12749424	282127	ZFP36	zinc finger protein 36, C3H type, homolog (mouse)	**3.1**	0.001767	0.151
	12834336	531389	FOSL1	FOS-like antigen 1	**3.0**	0.011003	0.155
	12730037	617470	IL18BP	interleukin 18 binding protein	**3.0**	0.008331	0.151
	12713795	282643	LPLUNC1	von Ebner minor salivary gland protein	**3.0**	0.013798	0.165
	12678828	282713	NFKBIZ	nuclear factor of kappa light polypeptide gene enhancer in B-cells inhibitor, zeta	**3.0**	0.017777	0.178
	12890889	528877	NR4A3	nuclear receptor subfamily 4, group A, member 3	**3.0**	0.043652	0.231
	12794515	407771	CCR1	chemokine (C-C motif) receptor 1	**2.9**	0.041164	0.223
	12885817	522921	HBEGF	heparin-binding EGF-like grow th factor	**2.9**	0.015022	0.170
	12780177	281356	NPPC	natriuretic peptide precursor C	**2.9**	0.010755	0.155
	12864151	282358	SLC2A3	solute carrier family 2 (facilitated glucose transporter), member 3	**2.9**	0.012611	0.161
	12760916	282081	SOCS3	suppressor of cytokine signaling 3	**2.9**	0.011989	0.159
	12733548	616239	TCN1	transcobalamin I (vitamin B12 binding protein, R binder family)	**2.9**	0.029497	0.194
	12756039	404071	MT1A	metallothionein 1E	**2.9**	0.008363	0.151
	12761264	286820	ALOX15B	arachidonate 15-lipoxygenase, type B	**2.8**	0.007283	0.151
	12835715	280686	F3	coagulation factor III (thromboplastin, tissue factor)	**2.8**	0.006610	0.151
	12738469	281246	IL10	interleukin 10	**2.8**	0.019142	0.178
	12718118	282431	PROKR2	prokineticin receptor 2	**2.8**	0.029952	0.194
	12898561	539960	STX11	syntaxin 11	**2.8**	0.009100	0.151
	12819271	507061	DUSP5	similar to dual specificity phosphatase 5	**2.7**	0.018607	0.178
	12875758	616115	NFKB1	nuclear factor of kappa light polypeptide gene enhancer in B-cells 1	**2.7**	0.017006	0.175
	12900295	515854	SGK1	serum/glucocorticoid regulated kinase 1	**2.7**	0.005203	0.151
	12714026	504492	SNAI1	snail homolog 1 (Drosophila)	**2.7**	0.003076	0.151
	12855397	538861	STEAP4	STEAP family member 4	**2.7**	0.006414	0.151
	12721602	281678	CEBPD	CCAAT/enhancer binding protein (C/EBP), delta	**2.6**	0.002454	0.151
	12749590	286824	HAS3	hyaluronan synthase 3	**2.6**	0.012791	0.162
	12736300	523618	IER5	immediate early response 5	**2.6**	0.008971	0.151
	12814908	100140740	IGSF6	immunoglobulin superfamily, member 6	**2.6**	0.030616	0.195
	12807296	100296226	LOC100296226	similar to phorbol-12-myristate-13-acetate-induced protein 1	**2.6**	0.007304	0.151
	12906283	790984	MIR222	microRNA mir-222	**2.6**	0.011069	0.155
	12682704	782774	P2RY13	purinergic receptor P2Y, G-protein coupled, 13	**2.6**	0.025733	0.192
	12738550	281485	SELL	selectin L	**2.6**	0.029807	0.194
	12756430	507167	CEACAM6	similar to carcinoembryonic antigen-related cell adhesion molecule 1	**2.6**	0.029255	0.194
	12842594	281094	CSF1	colony stimulating factor 1 (macrophage)	**2.6**	0.002257	0.151
	12842734	282118	VCAM1	vascular cell adhesion molecule 1	**2.6**	0.013758	0.165
	12818707	100139670	IFIT1	interferon-induced protein w ith tetratricopeptide repeats 1	**2.5**	0.016953	0.175
	12739170	509732	PDPN	podoplanin	**2.5**	0.007296	0.151
	12796519	537051	ADAMTS9	ADAM metallopeptidase w ith thrombospondin type 1 motif, 9	**2.4**	0.046766	0.237
	12705036	509889	FOSL2	FOS-like antigen 2	**2.4**	0.018607	0.178
	12687797	100313119	MIR2292	microRNA mir-2292	**2.4**	0.002746	0.151
	12825057	510037	RNF122	ring finger protein 122	**2.4**	0.041163	0.223
	12775981	509774	RNF19B	ring finger protein 19B	**2.4**	0.016803	0.175

	12718768	508133	SDC4	syndecan 4	**2.4**	0.006572	0.151
	12803236	539635	FAM65B	family with sequence similarity 65, member B	**2.4**	0.037503	0.217
	12749089	100336576	LOC100336576	cationic amino acid transporter 3-like	**2.4**	0.022193	0.187
	12762310	508666	LOC508666	similar to C-C motif chemokine 23 precursor	**2.4**	0.046056	0.236
	12700012	514626	MXD1	MAX dimerization protein 1	**2.4**	0.025190	0.192
	12878613	510803	PGLYRP2	peptidoglycan recognition protein 2	**2.4**	0.018354	0.178
	12899456	281496	SOD2	superoxide dismutase 2, mitochondrial	**2.4**	0.004611	0.151
	12745324	281534	TLR2	toll-like receptor 2	**2.4**	0.012428	0.160
	12895070	540664	TNC	tenascin C	**2.4**	0.006939	0.151
	12725414	521857	TRIB1	tribbles homolog 1 (Drosophila)	**2.4**	0.003350	0.151
	12798575	506945	BHLHB2	basic helix-loop-helix domain containing, class B, 2	**2.3**	0.003616	0.151
	12725595	529552	CYP7B1	cytochrome P450, family 7, subfamily B, polypeptide 1	**2.3**	0.008632	0.151
	12758131	526736	KLK10	kallikrein-related peptidase 10	**2.3**	0.037003	0.215
	12872030	538751	AREG	amphiregulin	**2.3**	0.042469	0.229
	12859830	517887	CYTH4	cytohesin 4	**2.3**	0.010653	0.155
	12708750	100170926	MIR19A	microRNA mir-19a	**2.3**	0.011531	0.157
	12724848	511077	MYC	v-myc myelocytomatosis viral oncogene homolog (avian)	**2.3**	0.015007	0.170
	12815192	281345	NCF1	neutrophil cytosolic factor 1	**2.3**	0.018547	0.178
	12824753	281407	PLAT	plasminogen activator, tissue	**2.3**	0.017568	0.177
	12683452	282258	IFNAR2	interferon (alpha, beta and omega) receptor 2	**2.2**	0.007083	0.151
	12906281	790983	MIR221	microRNA mir-221	**2.2**	0.035108	0.208
	12682933	100170919	MIRLET7C	microRNA let-7c	**2.2**	0.015582	0.172
	12866799	528390	NR4A1	nuclear receptor subfamily 4, group A, member 1	**2.2**	0.027291	0.193
	12790904	538896	BDKRB2	bradykinin receptor B2	**2.2**	0.013620	0.165
	12737028	539364	BTG2	BTG family, member 2	**2.2**	0.005730	0.151
	12739756	518609	CD55	CD55 molecule, decay accelerating factor for complement (Cromer blood group)	**2.2**	0.015385	0.171
	12738397	280687	F5	coagulation factor V (proaccelerin, labile factor)	**2.2**	0.033967	0.206
	12760305	100140276	LOC100140276	similar to jumonji domain containing 3	**2.2**	0.029107	0.194
	12889469	506097	NFIL3	nuclear factor, interleukin 3 regulated	**2.2**	0.023107	0.192
	12820145	526392	NFKB2	nuclear factor of kappa light polypeptide gene enhancer in B-cells 2 (p49/p100)	**2.2**	0.013738	0.165
	12901108	538384	PRDM1	PR domain containing 1, with ZNF domain	**2.2**	0.031866	0.199
	12711837	515333	ABCC4	ATP-binding cassette, sub-family C (CFTR/MRP), member 4	**2.1**	0.009585	0.151
	12832069	767970	ASRGL1	asparaginase like 1	**2.1**	0.008455	0.151
	12799059	515034	IRAK2	interleukin-1 receptor-associated kinase 2	**2.1**	0.003064	0.151
	12752468	527020	LOC527020	similar to Immunoglobulin superfamily member 1 precursor	**2.1**	0.008244	0.151
	12915093	297468078	LOC786591	PREDICTED: Bos taurus Rho-type GTPase-activating protein FLJ32810 (LOC786591), partial mRNA.	**2.1**	0.025023	0.192
	12749751	493645	C5AR1	complement component 5a receptor 1	**2.1**	0.047264	0.238
	12820137	526279	CASP7	caspase 7, apoptosis-related cysteine peptidase	**2.1**	0.029331	0.194
	12816039	507988	CYP3A4	cytochrome P450, subfamily IIIA, polypeptide 4	**2.1**	0.021879	0.186
	12768239	506088	ICAM2	intercellular adhesion molecule 2	**2.1**	0.028363	0.194
	12686272	539334	IL1RAP	interleukin 1 receptor accessory protein	**2.1**	0.016330	0.174
	12888608	791052	MIR24-2	microRNA mir-24-2	**2.1**	0.047939	0.239
	12681965	539640	RCAN1	regulator of calcineurin 1	**2.1**	0.012475	0.160
	12752266	522670	RELB	v-rel reticuloendotheliosis viral oncogene homolog B	**2.1**	0.007743	0.151

	12837291	508183	SLAMF6	SLAM family member 6	**2.1**	0.026100	0.192
	12893218	509167	CCL19	chemokine (C-C motif) ligand 19	**2.0**	0.013000	0.163
	12855777	615201	CLEC5A	C-type lectin domain family 5, member A	**2.0**	0.018371	0.178
	12780359	282851	DHRS9	dehydrogenase/reductase (SDR family) member 9	**2.0**	0.028307	0.194
	12915197	297468753	LOC783052	PREDICTED: Bos taurus elastin microfibril interfacer 2-like (LOC783052), partial mRNA.	**2.0**	0.047671	0.238
	12711686	510187	MGC155285	Uncharacterized protein C13orf33 homolog	**2.0**	0.010773	0.155
	12759042	613358	MT1E	metallothionein 1E	**2.0**	0.009672	0.151
	12787023	286842	BASP1	brain abundant, membrane attached signal protein 1	**2.0**	0.018175	0.178
	12843223	505040	CD53	CD53 molecule	**2.0**	0.010471	0.154
	12678891	414345	CD86	CD86 molecule	**2.0**	0.005445	0.151
	12845740	528076	DARC	Duffy blood group, chemokine receptor	**2.0**	0.023127	0.192
	12788556	784765	GAPT	GRB2-binding adaptor protein, transmembrane	**2.0**	0.021340	0.186
	12827463	100297981	LOC100297981	similar to early grow th response 2 protein	**2.0**	0.007332	0.151
	12914747	297462451	LOC100335170	PREDICTED: Bos taurus hypothetical protein LOC100335170 (LOC100335170), mRNA.	**2.0**	0.009782	0.151
	12749684	407171	LOC407171	Fc gamma 2 receptor	**2.0**	0.023581	0.192
	12906581	100313062	MIR505	microRNA mir-505	**2.0**	0.026934	0.193
	12776788	518283	PTAFR	platelet-activating factor receptor	**2.0**	0.036786	0.214
	12769871	512652	SSH2	slingshot homolog 2 (Drosophila)	**2.0**	0.016513	0.174
	12843460	506550	TSPAN1	tetraspanin 1	**2.0**	0.038474	0.219
	12726897	338039	CASP4	caspase 4, apoptosis-related cysteine peptidase	**2.0**	0.010413	0.154
	12875710	615107	CXCL10	chemokine (C-X-C motif) ligand 10	**2.0**	0.031821	0.199
	12711253	407230	HTR2A	5-hydroxytryptamine (serotonin) receptor 2A	**2.0**	0.014279	0.168
	12819421	509678	IFIT3	interferon-induced protein w ith tetratricopeptide repeats 3	**2.0**	0.018478	0.178
	12682289	613977	LOC613977	similar to TPA-induced transmembrane protein	**2.0**	0.011121	0.155
	12724561	507407	MSC	musculin (activated B-cell factor-1)	**2.0**	0.025580	0.192
	12875956	767910	PLAC8	placenta-specific 8	**2.0**	0.008982	0.151
	12740535	538050	RAB7B	RAB7B, member RAS oncogene family	**2.0**	0.009935	0.152
	12894962	539123	S1PR3	sphingosine-1-phosphate receptor 3	**2.0**	0.004260	0.151
	12738745	504407	SPSB1	splA/ryanodine receptor domain and SOCS box containing 1	**2.0**	0.033056	0.203
	12864466	504244	TUBA1C	tubulin, alpha 1c	**2.0**	0.028718	0.194
	12681567	534910	CD200	CD200 molecule	**1.9**	0.016168	0.174
	12821748	506132	CH25H	cholesterol 25-hydroxylase	**1.9**	0.019638	0.181
	12877406	407125	EGR1	early grow th response 1	**1.9**	0.045293	0.234
	12902104	100299249	LOC100299249	similar to interleukin 3 receptor, alpha	**1.9**	0.023798	0.192
	12888604	790986	MIR27A	microRNA mir-27a	**1.9**	0.039974	0.220
	12842051	100124505	PDE4B	phosphodiesterase 4B, cAMP-specific (phosphodiesterase E4 dunce homolog, Drosophila)	**1.9**	0.019818	0.182
	12841505	618250	S100A14	S100 calcium binding protein A14	**1.9**	0.010359	0.154
	12909544	536628	ACSL4	acyl-CoA synthetase long-chain family member 4	**1.9**	0.007083	0.151
	12819631	514159	ACSL5	acyl-CoA synthetase long-chain family member 5	**1.9**	0.013692	0.165
	12682216	541054	BTG3	BTG family, member 3	**1.9**	0.018422	0.178
	12855706	613798	C4H7orf53	chromosome 7 open reading frame 53 ortholog	**1.9**	0.021630	0.186
	12843976	509609	CD101	CD101 molecule	**1.9**	0.034619	0.207
	12687887	280795	FOS	FBJ murine osteosarcoma viral oncogene homolog	**1.9**	0.042286	0.229
	12786143	535347	FYB	FYN binding protein (FYB-120/130)	**1.9**	0.011657	0.157

	12680419	514613	MCM2	cyclin L1	**1.9**	0.021840	0.186
	12705580	513723	RALGDS	ral guanine nucleotide dissociation stimulator	**1.9**	0.005371	0.151
	12696054	533219	ACTC1	actin, alpha, cardiac muscle 1	**1.9**	0.038968	0.219
	12909023	529689	ATP11C	ATPase, class VI, type 11C	**1.9**	0.007564	0.151
	12741388	768081	LOC768081	hypothetical protein LOC768081	**1.9**	0.025752	0.192
	12805054	506727	NFKBIE	nuclear factor of kappa light polypeptide gene enhancer in B-cells inhibitor, epsilon	**1.9**	0.009494	0.151
	12778619	540245	NR4A2	nuclear receptor subfamily 4, group A, member 2	**1.9**	0.034389	0.206
	12885092	512682	SBNO2	strawberry notch homolog 2 (Drosophila)	**1.9**	0.049993	0.243
	12853224	360007	TFPI2	tissue factor pathway inhibitor 2	**1.9**	0.004930	0.151
	12683618	407131	CD80	CD80 molecule	**1.8**	0.017116	0.176
	12678583	281148	ETS2	v-ets erythroblastosis virus E26 oncogene homolog 2 (avian)	**1.8**	0.014558	0.170
	12712638	540287	GPR183	Epstein-Barr virus induced gene 2 (lymphocyte-specific G protein-coupled receptor)	**1.8**	0.009766	0.151
	12886630	533635	MCTP1	multiple C2 domains, transmembrane 1	**1.8**	0.001617	0.151
	12678533	280873	MX2	MX dynamin-like GTPase 2	**1.8**	0.014744	0.170
	12803191	538730	NRN1	neuritin 1	**1.8**	0.001552	0.151
	12868450	615588	RASSF9	Ras association (RalGDS/AF-6) domain family (N-terminal) member 9	**1.8**	0.004636	0.151
	12817048	518795	SOCS1	suppressor of cytokine signaling 1	**1.8**	0.008267	0.151
	12890269	514357	UGCG	UDP-glucose ceramide glucosyltransferase	**1.8**	0.026207	0.192
	12790583	532119	BDKRB1	bradykinin receptor B1	**1.8**	0.037240	0.216
	12799096	515406	CSRNP1	cysteine-serine-rich nuclear protein 1	**1.8**	0.016422	0.174
	12843892	508941	CYR61	cysteine-rich, angiogenic inducer, 61	**1.8**	0.025097	0.192
	12731455	508009	FLJ32810	hypothetical protein LOC508009	**1.8**	0.023341	0.192
	12781974	520327	KYNU	kynureninase (L-kynurenine hydrolase)	**1.8**	0.012523	0.160
	12759088	614198	NFKBID	nuclear factor of kappa light polypeptide gene enhancer in B-cells inhibitor, delta	**1.8**	0.044307	0.232
	12685668	533491	RASA2	RAS p21 protein activator 2	**1.8**	0.016351	0.174
	12895180	540944	TRAF1	TNF receptor-associated factor 1	**1.8**	0.031597	0.199
	12697278	616885	FGF7	fibroblast growth factor 7 (keratinocyte growth factor)	**1.8**	0.015795	0.172
	12712708	540990	LCP1	lymphocyte cytosolic protein 1 (L-plastin)	**1.8**	0.015114	0.170
	12710872	790980	MIR18A	microRNA mir-18a	**1.8**	0.032774	0.203
	12791243	617667	SERPINA3	serpin peptidase inhibitor, clade A (alpha-1 antiproteinase, antitrypsin), member 3	**1.8**	0.037845	0.218
	12848811	280714	AHR	aryl hydrocarbon receptor	**1.7**	0.016327	0.174
	12789146	338048	CYP11A1	cytochrome P450, family 11, subfamily A, polypeptide 1	**1.7**	0.010780	0.155
	12769517	511119	GJC1	gap junction protein, gamma 1, 45kDa	**1.7**	0.011878	0.158
	12823417	100313190	MIR2399	microRNA mir-2399	**1.7**	0.008186	0.151
	12678517	280872	MX1	myxovirus (influenza virus) resistance 1, interferon-inducible protein p78 (mouse)	**1.7**	0.024863	0.192
	12802355	520625	SLC44A4	solute carrier family 44, member 4	**1.7**	0.029495	0.194
	12680283	514194	CP	ceruloplasmin (ferroxidase)	**1.7**	0.000089	0.151
	12810727	783354	HRH4	histamine receptor H4	**1.7**	0.008284	0.151
	12698826	407221	IL18R1	interleukin 18 receptor 1	**1.7**	0.026065	0.192
	12863513	100294723	LOC100294723	similar to killer cell lectin-like receptor subfamily F, member 1	**1.7**	0.011273	0.155
	12784976	100313463	MIR2359	microRNA mir-2359	**1.7**	0.016427	0.174
	12886881	535277	TPM4	tropomyosin 4	**1.7**	0.011260	0.155
	12793470	538517	ARRDC4	arrestin domain containing 4	**1.7**	0.023206	0.192
	12883457	338066	CD97	CD97 molecule	**1.7**	0.014706	0.170

	12915097	297468145	LOC100335165	PREDICTED: Bos taurus hypothetical protein LOC100335165 (LOC100335165), mRNA.	**1.7**	0.014106	0.167
	12837806	511531	LOC511531	similar to guanylate binding protein 1	**1.7**	0.009123	0.151
	12911337	119891547	LOC614268	PREDICTED: Bos taurus seminal vesicle antigen-like 1-like (LOC614268), mRNA.	**1.7**	0.046864	0.237
	12888606	791041	MIR23A	microRNA mir-23a	**1.7**	0.030058	0.194
	12857324	317710	PLBD1	phospholipase B domain containing 1	**1.7**	0.045269	0.234
	12855928	616414	RGS2	regulator of G-protein signaling 2, 24kDa	**1.7**	0.013416	0.165
	12685288	527910	SKIL	SKI-like oncogene	**1.7**	0.027542	0.193
	12846359	534256	CLCA2	chloride channel accessory 2	**1.6**	0.033120	0.203
	12761205	282642	ITGB3	integrin, beta 3 (platelet glycoprotein IIIa, antigen CD61)	**1.6**	0.005634	0.151
	12722742	522795	KLF10	Kruppel-like factor 10	**1.6**	0.005602	0.151
	12717764	100170928	MIR103-2	microRNA mir-103-2	**1.6**	0.006006	0.151
	12739190	510169	MYBPH	myosin binding protein H	**1.6**	0.019638	0.181
	12730744	281249	IL18	interleukin 18 (interferon-gamma-inducing factor)	**1.6**	0.023955	0.192
	12914989	297466658	LOC100335932	PREDICTED: Bos taurus tec protein tyrosine kinase (LOC100335932), mRNA.	**1.6**	0.015052	0.170
	12898795	613678	LOC613678	similar to cleavage stimulation factor subunit 3	**1.6**	0.021047	0.185
	12901502	782472	LOC782472	hypothetical LOC782472	**1.6**	0.038410	0.219
	12910548	784704	LOC784704	similar to DnaJ (Hsp40) homolog, subfamily A, member 1	**1.6**	0.004495	0.151
	12869241	784769	LOC784769	similar to MGC127725 protein	**1.6**	0.009198	0.151
	12737877	791042	MIR29B-2	microRNA mir-29b-2	**1.6**	0.009665	0.151
	12746932	519922	OAS1	2',5'-oligoadenylate synthetase 1, 40/46kDa	**1.6**	0.003084	0.151
	12853718	508836	SNX10	sorting nexin 10	**1.6**	0.041157	0.223
	12709067	282102	TRPC4	transient receptor potential cation channel, subfamily C, member 4	**1.6**	0.043375	0.231
	12873221	281564	UGDH	UDP-glucose dehydrogenase	**1.6**	0.026566	0.192
	12684815	516536	C1H21ORF63	chromosome 21 open reading frame 63 ortholog	**1.6**	0.026117	0.192
	12899452	281193	GJA1	gap junction protein, alpha 1, 43kDa	**1.6**	0.029725	0.194
	12849229	282879	HGF	hepatocyte growth factor (hepapoietin A; scatter factor)	**1.6**	0.013365	0.165
	12756340	505591	LOC505591	similar to LOC618035 protein	**1.6**	0.030146	0.194
	12693564	282329	PTGER2	prostaglandin E receptor 2 (subtype EP2), 53kDa	**1.6**	0.002614	0.151
	12723360	540195	RNF19A	ring finger protein 19A	**1.6**	0.036067	0.212
	12761636	504960	CD68	CD68 molecule	**1.6**	0.018767	0.178
	12732540	527762	KCNE3	potassium voltage-gated channel, Isk-related family, member 3	**1.6**	0.046914	0.237
	12915081	297467992	LOC100294770	PREDICTED: Bos taurus hypothetical LOC100294770 (LOC100294770), mRNA.	**1.6**	0.043652	0.231
	12915137	297468411	LOC100335378	PREDICTED: Bos taurus hypothetical protein LOC100335378 (LOC100335378), mRNA.	**1.6**	0.043652	0.231
	12915359	297469511	LOC100335892	PREDICTED: Bos taurus hypothetical protein LOC100335892 (LOC100335892), mRNA.	**1.6**	0.043652	0.231
	12896461	100336955	LOC100336955	Monofunctional C1-tetrahydrofolate synthase, mitochondrial-like	**1.6**	0.015884	0.173
	12850240	515156	STEAP1	six transmembrane epithelial antigen of the prostate 1	**1.6**	0.047459	0.238
	12902909	282092	TIMP1	TIMP metallopeptidase inhibitor 1	**1.6**	0.010909	0.155
	12680034	512171	ADAMTS1	ADAM metallopeptidase with thrombospondin type 1 motif, 1	**1.5**	0.000943	0.151
	12915559	61316285	BOLA-DQA2	Bos taurus major histocompatibility complex, class II, DQ alpha 2 (BOLA-DQA2), mRNA.	**1.5**	0.015724	0.172
	12862774	767905	GLIPR1	GLI pathogenesis-related 1	**1.5**	0.025953	0.192
	12910555	784938	LDHA	lactate dehydrogenase A	**1.5**	0.005066	0.151
	12888844	100313011	MIR24-1	microRNA mir-24-1	**1.5**	0.039274	0.219
	12809644	513825	SERPINB8	serpin peptidase inhibitor, clade B (ovalbumin), member 8	**1.5**	0.026453	0.192
	12707468	539470	GPR75	G protein-coupled receptor 75	**1.5**	0.039780	0.219

	12759047	613446	PLLP	plasma membrane proteolipid (plasmolipin)	**-1.5**	0.039750	0.219
	12847127	540995	ST6GALNAC5	ST6 (alpha-N-acetyl-neuraminyl-2,3-beta-galactosyl-1,3)-N-acetylgalactosaminide alpha-2,6-sialyltransferase 5	**-1.5**	0.036645	0.214
	12747950	614062	CCDC60	coiled-coil domain containing 60	**-1.5**	0.046933	0.237
	12729100	534401	ZBTB16	zinc finger and BTB domain containing 16	**-1.5**	0.021071	0.185
	12708472	783020	SULT1C2	sulfotransferase family, cytosolic, 1C, member 2	**-1.6**	0.027613	0.193
	12855679	613658	TMEM195	transmembrane protein 195	**-1.6**	0.028955	0.194
	12886948	537062	SLC27A6	solute carrier family 27 (fatty acid transporter), member 6	**-1.6**	0.038867	0.219
	12905967	767883	CCDC160	coiled-coil domain containing 160	**-1.6**	0.019244	0.179
	12773218	768225	NR1D1	nuclear receptor subfamily 1, group D, member 1	**-1.6**	0.020260	0.184
	12801577	509275	HIST1H1D	histone cluster 1, H1d	**-1.7**	0.014043	0.167
	12896448	100336854	LOC100336854	hypothetical protein LOC100336854	**-1.7**	0.000812	0.151
	12768497	507464	KRT13	keratin 13	**-1.8**	0.015580	0.172
	12713066	781119	SLITRK6	SLIT and NTRK-like family, member 6	**-1.8**	0.026755	0.192
	12705776	515213	CLIP4	CAP-GLY domain containing linker protein family, member 4	**-1.8**	0.006037	0.151
	12765513	616622	PRR15L	proline rich 15-like	**-2.0**	0.002749	0.151
***S. aureus* 1 h**							
	12721560	100313355	MIR2310	microRNA mir-2310	**1.9**	0.004641	0.805
	12875710	615107	CXCL10	chemokine (C-X-C motif) ligand 10	**1.8**	0.044207	0.805
	12767029	281044	CCL8	chemokine (C-C motif) ligand 8	**1.7**	0.011280	0.805
	12786931	281358	NPR3	natriuretic peptide receptor C/guanylate cyclase C (atrionatriuretic peptide receptor C)	**1.7**	0.015105	0.805
	12721564	100336590	LOC100336590	thyroglobulin-like	**1.7**	0.018286	0.805
	12847923	781576	LOC781576	similar to ribosomal protein L35a	**-1.5**	0.034415	0.805
	12855679	613658	TMEM195	transmembrane protein 195	**-1.5**	0.049349	0.805
	12873056	100337111	LOC100337111	T-cell activation Rho GTPase-activating protein-like	**-1.6**	0.016877	0.805
***S. aureus* 2 h**							
	12678533	280873	MX2	MX dynamin-like GTPase 2	**1.6**	0.012689	1.000
***S. aureus* 3 h**							
	12820239	529092	LOC529092	similar to RIKEN cDNA 9930023K05	**3.0**	0.048793	0.338
	12767029	281044	CCL8	chemokine (C-C motif) ligand 8	**2.4**	0.048360	0.338
	12832640	281474	SAA3	serum amyloid A 3	**2.3**	0.045092	0.338
	12703851	281595	ACTG2	actin, gamma 2, smooth muscle, enteric	**2.0**	0.038055	0.338
	12914065	194685682	LOC535409	PREDICTED: Bos taurus hCG1642212-like (LOC535409), mRNA.	**2.0**	0.006817	0.338
	12900966	534650	RSPO3	R-spondin 3 homolog (Xenopus laevis)	**2.0**	0.010822	0.338
	12741898	280753	CPE	carboxypeptidase E	**1.9**	0.013224	0.338
	12723184	535166	LOC535166	similar to mKIAA1077 protein	**1.9**	0.026455	0.338
	12804428	280943	TNF	tumor necrosis factor (TNF superfamily, member 2)	**1.9**	0.026503	0.338
	12682595	767844	B3GALNT1	beta-1,3-N-acetylgalactosaminyltransferase 1 (globoside blood group)	**1.9**	0.023814	0.338
	12786020	531659	C1QTNF3	C1q and tumor necrosis factor related protein 3	**1.9**	0.033280	0.338
	12726800	281941	NCAM1	neural cell adhesion molecule 1	**1.9**	0.013062	0.338
	12680435	514701	LOC514701	similar to Protein FAM3B precursor (Cytokine-like protein 2-21)	**1.8**	0.029124	0.338
	12710796	781586	SLITRK5	SLIT and NTRK-like family, member 5	**1.8**	0.004200	0.338

	12718011	281528	TGM2	transglutaminase 2 (C polypeptide, protein-glutamine-gamma-glutamyltransferase)	**1.8**	0.004554	0.338
	12805551	514076	CFB	complement factor B	**1.8**	0.036931	0.338
	12875470	540340	SYNPO2	synaptopodin 2	**1.8**	0.019635	0.338
	12787470	514739	MAP1B	microtubule-associated protein 1B	**1.8**	0.023210	0.338
	12892875	497015	TPM2	tropomyosin 2 (beta)	**1.8**	0.034397	0.338
	12791970	507093	ALDH1A3	aldehyde dehydrogenase 1 family, member A3	**1.7**	0.041459	0.338
	12793557	539332	CFL2	cofilin 2 (muscle)	**1.7**	0.021999	0.338
	12880668	534583	CNN1	calponin 1, basic, smooth muscle	**1.7**	0.044050	0.338
	12820017	523601	EMX2	empty spiracles homeobox 2	**1.7**	0.005488	0.338
	12899452	281193	GJA1	gap junction protein, alpha 1, 43kDa	**1.7**	0.043797	0.338
	12710121	533449	ITGBL1	integrin, beta-like 1 (with EGF-like repeat domains)	**1.7**	0.026166	0.338
	12885365	514246	JUNB	jun B proto-oncogene	**1.7**	0.045692	0.338
	12702463	613877	MEIS1	Meis homeobox 1	**1.7**	0.028333	0.338
	12786931	281358	NPR3	natriuretic peptide receptor C/guanylate cyclase C (atrionatriuretic peptide receptor C)	**1.7**	0.017933	0.338
	12713599	281426	PRND	prion protein 2 (dublet)	**1.7**	0.010792	0.338
	12679533	509461	TMEM45A	transmembrane protein 45A	**1.7**	0.035010	0.338
	12725810	538634	CTHRC1	collagen triple helix repeat containing 1	**1.7**	0.019286	0.338
	12699298	507311	DYNC2LI1	dynein, cytoplasmic 2, light intermediate chain 1	**1.7**	0.049068	0.338
	12698536	281860	IL1RN	interleukin 1 receptor antagonist	**1.7**	0.047083	0.338
	12899396	100337123	LOC100337123	R-spondin 3-like	**1.7**	0.047498	0.338
	12905207	537655	LOC537655	similar to dystrophin	**1.7**	0.008984	0.338
	12696694	539573	MEIS2	Meis homeobox 2	**1.7**	0.044435	0.338
	12876527	100313363	MIR143	microRNA mir-143	**1.7**	0.047924	0.338
	12681397	533642	PLOD2	procollagen-lysine, 2-oxoglutarate 5-dioxygenase 2	**1.7**	0.039214	0.338
	12902773	100337181	LOC100337181	p21-activated kinase 3-like	**1.7**	0.039205	0.338
	12862249	613907	BHLHE41	basic helix-loop-helix family, member e41	**1.6**	0.038092	0.338
	12707387	538967	CDC42EP3	CDC42 effector protein (Rho GTPase binding) 3	**1.6**	0.013384	0.338
	12875710	615107	CXCL10	chemokine (C-X-C motif) ligand 10	**1.6**	0.031607	0.338
	12726248	782927	LOC782927	hypothetical LOC782927	**1.6**	0.004579	0.338
	12774042	100313251	MIR2917	microRNA mir-2917	**1.6**	0.018368	0.338
	12892150	785045	PGM5	phosphoglucomutase 5	**1.6**	0.024338	0.338
	12852880	280904	PTN	pleiotrophin	**1.6**	0.005335	0.338
	12875211	535372	SGCB	sarcoglycan, beta (43kDa dystrophin-associated glycoprotein)	**1.6**	0.019657	0.338
	12840882	540527	EXTL2	exostoses (multiple)-like 2	**1.6**	0.017529	0.338
	12775947	509422	FKBP7	FK506 binding protein 7	**1.6**	0.016294	0.338
	12863952	281239	IGF1	insulin-like growth factor 1 (somatomedin C)	**1.6**	0.010843	0.338
	12730744	281249	IL18	interleukin 18 (interferon-gamma-inducing factor)	**1.6**	0.031250	0.338
	12793818	617436	ISLR2	immunoglobulin superfamily containing leucine-rich repeat 2	**1.6**	0.015846	0.338
	12914747	297462451	LOC100335170	PREDICTED: Bos taurus hypothetical protein LOC100335170 (LOC100335170), mRNA.	**1.6**	0.023742	0.338
	12915093	297468078	LOC786591	PREDICTED: Bos taurus Rho-type GTPase-activating protein FLJ32810 (LOC786591), partial mRNA.	**1.6**	0.037429	0.338
	12876752	280841	LOX	lysyl oxidase	**1.6**	0.015153	0.338
	12765581	617087	MXRA7	matrix-remodelling associated 7	**1.6**	0.032130	0.338
	12723845	788352	NKAIN3	Na+/K+ transporting ATPase interacting 3	**1.6**	0.010601	0.338
	12893422	511556	SLC46A2	solute carrier family 46, member 2	**1.6**	0.023371	0.338

	12873727	507537	SPARCL1	SPARC-like 1 (hevin)	**1.6**	0.019090	0.338
	12691699	541281	THBS4	thrombospondin 4	**1.6**	0.023810	0.338
	12893122	507990	ASPN	asporin	**1.6**	0.038998	0.338
	12781192	510833	COL3A1	collagen, type III, alpha 1	**1.6**	0.030587	0.338
	12747823	539524	HSPB8	heat shock 22kDa protein 8	**1.6**	0.027804	0.338
	12824632	789528	LOC789528	similar to zinc finger protein 385D	**1.6**	0.047038	0.338
	12786378	540230	MSX2	msh homeobox 2	**1.6**	0.040677	0.338
	12778611	540224	RND3	Rho family GTPase 3	**1.6**	0.013070	0.338
	12843025	407174	SLC6A17	solute carrier family 6, member 17	**1.6**	0.018419	0.338
	12804999	506107	BAG2	BCL2-associated athanogene 2	**1.6**	0.011536	0.338
	12710496	613449	DCLK1	doublecortin-like kinase 1	**1.6**	0.033150	0.338
	12870781	510906	DDIT4L	DNA-damage-inducible transcript 4-like	**1.6**	0.005242	0.338
	12742930	519602	IFT81	intraflagellar transport 81 homolog (Chlamydomonas)	**1.6**	0.035189	0.338
	12896224	100140348	LOC100140348	hypothetical protein LOC100140348	**1.6**	0.009755	0.338
	12726731	281445	RCN1	reticulocalbin 1, EF-hand calcium binding domain	**1.6**	0.011940	0.338
	12871689	534164	SLIT2	slit homolog 2 (Drosophila)	**1.6**	0.030279	0.338
	12779533	618122	SYNC	syncoilin, intermediate filament protein	**1.6**	0.046216	0.338
	12743502	532970	TBX5	T-box 5	**1.6**	0.045436	0.338
	12681567	534910	CD200	CD200 molecule	**1.5**	0.043276	0.338
	12877406	407125	EGR1	early growth response 1	**1.5**	0.013280	0.338
	12755625	280692	HP	haptoglobin	**1.5**	0.040887	0.338
	12698109	100299044	LOC100299044	similar to pleckstrin homology domain-containing, family A (phosphoinositide binding specific) member 2	**1.5**	0.007052	0.338
	12817585	530050	MYH11	myosin, heavy chain 11, smooth muscle	**1.5**	0.039766	0.338
	12683570	338037	MYLK	myosin light chain kinase	**1.5**	0.033152	0.338
	12870067	281972	PDE5A	phosphodiesterase 5A, cGMP-specific	**1.5**	0.008537	0.338
	12899131	100125240	PLN	phospholamban	**1.5**	0.036337	0.338
	12737194	540901	PRRX1	paired related homeobox 1	**1.5**	0.038937	0.338
	12682546	617797	SH3BGR	similar to SH3 domain-binding glutamic acid-rich protein (SH3BGR protein)	**1.5**	0.008907	0.338
	12898880	615490	SMOC2	SPARC related modular calcium binding 2	**1.5**	0.047716	0.338
	12681808	538564	COL8A1	collagen, type VIII, alpha 1	**1.5**	0.009741	0.338
	12727136	506627	DKK3	dickkopf homolog 3 (Xenopus laevis)	**1.5**	0.034911	0.338
	12907047	281165	FLNA	filamin A, alpha	**1.5**	0.045644	0.338
	12876894	281210	GPX3	glutathione peroxidase 3 (plasma)	**1.5**	0.038490	0.338
	12863526	100295249	LOC100295249	similar to homeobox C8	**1.5**	0.025696	0.338
	12787312	511595	PARP8	poly (ADP-ribose) polymerase family, member 8	**1.5**	0.026134	0.338
	12681843	539034	PFN2	profilin 2	**1.5**	0.038536	0.338
	12801427	507058	PI16	peptidase inhibitor 16	**1.5**	0.040434	0.338
	12893989	518308	PRUNE2	prune homolog 2 (Drosophila)	**1.5**	0.010941	0.338
	12846750	538209	PTGFRN	prostaglandin F2 receptor negative regulator	**1.5**	0.045605	0.338
	12710465	540806	SMAD9	SMAD family member 9	**1.5**	0.008021	0.338
	12724392	505317	TRPA1	transient receptor potential cation channel, subfamily A, member 1	**1.5**	0.004321	0.338
	12709067	282102	TRPC4	transient receptor potential cation channel, subfamily C, member 4	**1.5**	0.016720	0.338
	12808491	538792	ZNF521	zinc finger protein 521	**1.5**	0.028294	0.338
	12720208	530147	BAMBI	BMP and activin membrane-bound inhibitor homolog (Xenopus laevis)	**1.5**	0.042448	0.338

	12792596	516210	CKB	creatine kinase, brain	**1.5**	0.003840	0.338
	12764980	539361	SREBF1	sterol regulatory element binding transcription factor 1	**-1.5**	0.042383	0.338
	12773920	100295933	LOC100295933	similar to ArfGAP with SH3 domain, ankyrin repeat and PH domain 3	**-1.5**	0.041979	0.338
	12904797	529934	LOC529934	similar to Uncharacterized protein C18orf58	**-1.6**	0.019434	0.338
	12780150	280994	ALPL	alkaline phosphatase, liver/bone/kidney	**-1.6**	0.040981	0.338
	12789614	508724	FAH	fumarylacetoacetate hydrolase (fumarylacetoacetase)	**-1.6**	0.045138	0.338
	12791659	281799	GPR68	G protein-coupled receptor 68	**-1.6**	0.046706	0.338
	12693799	494548	DIO2	deiodinase, iodothyronine, type II	**-1.6**	0.038624	0.338
	12842026	790815	SCLY	selenocysteine lyase	**-1.6**	0.045954	0.338
	12832574	100336865	LOC100336865	spectrin, beta, non-erythrocytic 2-like	**-1.6**	0.038770	0.338
	12797432	783185	LOC783185	similar to light chain 3	**-1.7**	0.042449	0.338
	12776704	516133	FGR	Gardner-Rasheed feline sarcoma viral (v-fgr) oncogene homolog	**-1.7**	0.043701	0.338
	12791138	614707	SEMA7A	semaphorin 7A, GPI membrane anchor (John Milton Hagen blood group)	**-1.7**	0.047611	0.338
	12841874	786977	LOC786977	similar to ANKRD protein	**-1.7**	0.034392	0.338
	12847923	781576	LOC781576	similar to ribosomal protein L35a	**-1.8**	0.012739	0.338

Supplementary Table 2. List of oligonucleotide primers. Sequences of the oligonucleotide primers, the accession number of the relevant source sequence and the size of the amplificate are also indicated.

Gene	Ensembl no.	Primer sequence (5´–3´)	Amplificate size (bp)
TNF	ENSBTAG00000025471	CTTCTGCCTGCTGCACTTCG *<br>GAGTTGATGTCGGCTACAACG *	156
IL6	ENSBTAG00000014921	GGAGGAAAAGGACGGATGCT<br>GGTCAGTGTTTGTGGCTGGA	227
CXCL8	ENSBTAG00000019716	CCTCTTGTTCAATATGACTTCCA<br>GGCCCACTCTCAATAACTCTC	170
CCL20	ENSBTAG00000021326	CAGCAAGTCAGAAGCAAGCAA<br>CCCACTTCTTCTTTGGATCTGC	179
LAP	ENSBTAG00000027225	AGGCTCCATCACCTGCTCCTT<br>CCTGCAGCATTTTACTTGGGCT	182
S100A9	ENSBTAG00000006505	CTCAAACAGAAGGCGGGAAA<br>TTGTGTCCAGATCCTCCATGA	225
MX2	ENSBTAG00000008471	CACCTACCGCAACATTACGC<br>GCTGATGCCAAGTCCATTCC	103
LCN2	ENSBTAG00000014149	CCAACTACGAGCTGAAGGAAGAC<br>TGGGAGCTTGGGACAAAAGT	103

*upper line: forward-, lower line: reverse-primer

Supplementary Table 3. List of pathogen-species specific DEGs. List of DEGs having exclusively been detected during *E. coli* (3A) or *S. aureus* (3B) infection, sorted according to functional gene families. The fold change values (FC) and the relevant upstream regulators as identified by the Ingenuity software are also indicated. GPCR, G-protein coupled receptor; GF, growth factor; NR, nuclear receptor; TR, transcription regulator; TMR, transmembrane receptor.

A) Genes regulated only by *E. coli*

Factor family	Symbol	Gene	FC		Upstream regulator		
			2h	3h			
cytokine	CXCL2	C-X-C motif chemokine ligand 2	8,3	21,3	LPS	TNF	IL1B
cytokine	CCL20	C-C motif chemokine ligand 20	8,3	18,2	LPS	TNF	IL1B
cytokine	CXCL8	C-X-C motif chemokine ligand 8	9,1	17,9	LPS	TNF	IL1B
cytokine	IL6	interleukin 6	14,5	15,4	LPS	TNF	IL1B
cytokine	IL1B	interleukin 1 beta	11,2	13,9	LPS	TNF	IL1B
cytokine	CCL2	C-C motif chemokine ligand 2	11,0	13,7	LPS	TNF	IL1B
cytokine	IL1A	interleukin 1 alpha	7,7	12,0	LPS	TNF	IL1B
cytokine	LIF	leukemia inhibitory factor	4,5	9,1	LPS	TNF	IL1B
cytokine	TNF	tumor necrosis factor	3,4	8,3			
cytokine	CXCL5	C-X-C motif chemokine ligand 5	4,5	5,6	LPS	TNF	IL1B
cytokine	CCL4	C-C motif chemokine ligand 4	3,3	5,0	LPS	TNF	IL1B
cytokine	CSF3	colony stimulating factor 3	2,5	3,3	LPS	TNF	IL1B
cytokine	IL10	interleukin 10		2,8	LPS	TNF	IL1B
cytokine	CSF1	colony stimulating factor 1	2,2	2,6	LPS	TNF	IL1B
cytokine	CCL19	C-C motif chemokine ligand 19	1,6	2,0	LPS	TNF	
cytokine	TIMP1	TIMP metallopeptidase inhibitor 1		1,6	LPS	TNF	IL1B
enzyme	RND1	Rho family GTPase 1	9,1	12,3		TNF	
enzyme	PTGS2	prostaglandin-endoperoxide synthase 2	5,6	8,3	LPS	TNF	IL1B
enzyme	TNFAIP3	TNF alpha induced protein 3	3,7	5,0	LPS	TNF	IL1B
enzyme	GFPT2	glutamine-fructose-6-phosphate transaminase 2	5,0	4,5		TNF	
enzyme	ACOD1	aconitate decarboxylase 1	3,6	4,3			
enzyme	ZC3H12A	zinc finger CCCH-type containing 12A	2,4	3,6		TNF	IL1B

enzyme	BIRC3	baculoviral IAP repeat containing 3	2,8	3,3	LPS	TNF	IL1B
enzyme	CD274	CD274 molecule	2,8	3,2	LPS	TNF	IL1B
enzyme	LYZ	lysozyme	3,0	3,1	LPS		
enzyme	ALOX15B	arachidonate 15-lipoxygenase, type B	2,0	2,8		TNF	
enzyme	STEAP4	STEAP4 metalloreductase	2,1	2,7	LPS	TNF	
enzyme	HAS3	hyaluronan synthase 3	2,6	2,6			
enzyme	SOD2	superoxide dismutase 2, mitochondrial	1,9	2,4	LPS	TNF	IL1B
enzyme	CYP7B1	cytochrome P450 family 7 subfamily B member 1	2,0	2,3	LPS	TNF	
enzyme	NCF1	neutrophil cytosolic factor 1		2,3	LPS	TNF	
enzyme	F5	coagulation factor V		2,2			
enzyme	CYP3A4	cytochrome P450 family 3 subfamily A member 4		2,1	LPS		IL1B
enzyme	CH25H	cholesterol 25-hydroxylase		1,9	LPS	TNF	
enzyme	PDE4B	phosphodiesterase 4B	1,7	1,9	LPS	TNF	IL1B
enzyme	ACSL4	acyl-CoA synthetase long-chain family member 4		1,9			
enzyme	ACSL5	acyl-CoA synthetase long-chain family member 5		1,9			
enzyme	MCM2	minichromosome maintenance complex component 2	1,6	1,9			
enzyme	ACTC1	actin, alpha, cardiac muscle 1		1,9	LPS		
enzyme	UGCG	UDP-glucose ceramide glucosyltransferase		1,8		TNF	IL1B
enzyme	KYNU	kynureninase		1,8	LPS	TNF	
enzyme	CYP11A1	cytochrome P450 family 11 subfamily A member 1		1,7		TNF	IL1B
enzyme	MX1	MX dynamin like GTPase 1		1,7	LPS	TNF	IL1B
enzyme	CP	ceruloplasmin (ferroxidase)		1,7	LPS	TNF	IL1B
enzyme	ARG2	arginase 2	1,6	1,6	LPS		
enzyme	OAS1	2'-5'-oligoadenylate synthetase 1		1,6	LPS	TNF	
enzyme	UGDH	UDP-glucose 6-dehydrogenase		1,6			IL1B
enzyme	RNF19A	ring finger protein 19A, RBR E3 ubiquitin protein ligase		1,6	LPS		

enzyme	RAB27A	RAB27A, member RAS oncogene family	1,6	1,6			
enzyme	LDHA	lactate dehydrogenase A		1,5		TNF	IL1B
GPCR	GPR84	G protein-coupled receptor 84	5,0	7,7	LPS	TNF	
GPCR	CXCR1	C-X-C motif chemokine receptor 1	7,7	6,7	LPS	TNF	IL1B
GPCR	CXCR2	C-X-C motif chemokine receptor 2	3,8	4,0	LPS	TNF	
GPCR	CCR1	C-C motif chemokine receptor 1	2,6	2,9	LPS	TNF	IL1B
GPCR	BDKRB2	bradykinin receptor B2	1,7	2,2		TNF	
GPCR	C5AR1	complement component 5a receptor 1		2,1	LPS	TNF	
GPCR	ACKR1	atypical chemokine receptor 1 (Duffy blood group)	2,0	2,0			
GPCR	PTAFR	platelet activating factor receptor		2,0	LPS		
GPCR	S1PR3	sphingosine-1-phosphate receptor 3		2,0	LPS	TNF	
GPCR	BDKRB1	bradykinin receptor B1		1,8	LPS	TNF	IL1B
GPCR	ADGRE5	adhesion G protein-coupled receptor E5		1,7			
GPCR	PTGER2	prostaglandin E receptor 2		1,6	LPS		
GPCR	C3AR1	complement component 3a receptor 1	-1,6	-1,6	LPS		
GF	ESM1	endothelial cell specific molecule 1	2,6	4,5		TNF	
GF	HBEGF	heparin binding EGF like growth factor	2,1	2,9	LPS	TNF	IL1B
GF	AREG	amphiregulin		2,3	LPS		
GF	FGF7	fibroblast growth factor 7		1,8			IL1B
GF	BMP2	bone morphogenetic protein 2	1,6	1,6	LPS	TNF	IL1B
GF	HGF	hepatocyte growth factor		1,6	LPS	TNF	IL1B
ion channel	GJC1	gap junction protein gamma 1		1,7			
ion channel	CLCA2	chloride channel accessory 2		1,6			
ion channel	KCNE3	potassium voltage-gated channel subfamily E regulatory subunit 3		1,6			
kinase	MAP3K8	mitogen-activated protein kinase kinase kinase 8	3,2	3,6	LPS	TNF	IL1B
kinase	CDKN1A	cyclin-dependent kinase inhibitor 1A	2,9	3,3	LPS	TNF	IL1B
kinase	SGK1	serum/glucocorticoid regulated kinase 1	1,7	2,7		TNF	
kinase	TRIB1	tribbles pseudokinase 1	1,8	2,4	LPS		

kinase	IRAK2	interleukin 1 receptor associated kinase 2	1,6	2,1	LPS	TNF	IL1B
kinase	PIM1	Pim-1 proto-oncogene, serine/threonine kinase	1,6	1,6	LPS	TNF	IL1B
NR	NR4A3	NR subfamily 4 group A member 3	1,9	3,0	LPS	TNF	IL1B
NR	NR4A1	NR subfamily 4 group A member 1		2,2	LPS	TNF	IL1B
NR	NR4A2	NR subfamily 4 group A member 2		1,9	LPS	TNF	IL1B
NR	AHR	aryl hydrocarbon receptor		1,7	LPS		
NR	NR1D1	NR subfamily 1 group D member 1		-1,6			IL1B
microRNA	mir-223	microRNA 223	5,9	4,8			
microRNA	mir-21	microRNA 21	2,1	3,4			
microRNA	mir-221	microRNA 221	2,2	2,6			
microRNA	mir-19	microRNA 19a		2,3			
microRNA	let-7	microRNA let-7a-1		2,2			
microRNA	mir-24	microRNA 24-1		2,1			
microRNA	mir-27	microRNA 27a		1,9			
microRNA	mir-17	microRNA 17		1,8			
microRNA	mir-23	microRNA 23a		1,7			
microRNA	mir-103	microRNA 107		1,6			
other	S100A8	S100 calcium binding protein A8	7,7	7,7	LPS	TNF	IL1B
other	MEFV	Mediterranean fever	5,3	7,1	LPS	TNF	IL1B
other	S100A12	S100 calcium binding protein A12	7,1	7,1	LPS		
other	PLEK	pleckstrin	4,2	5,6	LPS		
other	ERRFI1	ERBB receptor feedback inhibitor 1	4,2	5,3			IL1B
other	NAIP	NLR family, apoptosis inhibitory protein	5,0	5,3		TNF	
other	MT2A	metallothionein 2A	5,0	5,0	LPS		IL1B
other	IER3	immediate early response 3	2,1	4,8	LPS	TNF	IL1B
other	TNFAIP2	TNF alpha induced protein 2	5,0	4,5	LPS	TNF	IL1B
other	S100A2	S100 calcium binding protein A2	4,3	4,3	LPS		
other	BCL2A1	BCL2 related protein A1	2,8	4,0	LPS	TNF	IL1B
other	SERPINE1	serpin family E member 1	2,9	4,0	LPS	TNF	IL1B

other	ICAM2	intercellular adhesion molecule 2	5,0	3,7	LPS	TNF	
other	SAMSN1	SAM domain, SH3 domain and nuclear localization signals 1	2,9	3,7	LPS		
other	RGS16	regulator of G-protein signaling 16	2,8	3,3	LPS	TNF	IL1B
other	S100A9	S100 calcium binding protein A9	3,8	3,3	LPS	TNF	IL1B
other	SRGN	serglycin	3,2	3,3			IL1B
other	NLRP3	NLR family, pyrin domain containing 3	1,9	3,1	LPS	TNF	
other	IL18BP	interleukin 18 binding protein	3,0	3,0		TNF	
other	NPPC	natriuretic peptide C	2,6	2,9	LPS		
other	MT1A	metallothionein 1A	2,8	2,9		TNF	
other	ANGPTL4	angiopoietin like 4	2,8	2,8	LPS	TNF	IL1B
other	PTX3	pentraxin 3	2,8	2,8	LPS	TNF	IL1B
other	IER5	immediate early response 5	2,2	2,6			
other	IFIT1	interferon induced protein with tetratricopeptide repeats 1	2,0	2,5	LPS	TNF	IL1B
other	PDPN	podoplanin	1,9	2,5		TNF	
other	SDC4	syndecan 4	2,2	2,4	LPS	TNF	IL1B
other	TNC	tenascin C		2,4	LPS	TNF	
other	CD55	CD55 molecule (Cromer blood group)	1,8	2,2	LPS	TNF	IL1B
other	CLEC5A	C-type lectin domain family 5 member A	1,6	2,0	LPS	TNF	
other	MT1E	metallothionein 1E	1,7	2,0			
other	CD53	CD53 molecule	1,6	2,0	LPS		
other	IFIT3	interferon induced protein with tetratricopeptide repeats 3	1,9	2,0	LPS	TNF	IL1B
other	PLAC8	placenta specific 8	1,7	2,0			
other	SPSB1	splA/ryanodine receptor domain and SOCS box containing 1	1,7	2,0		TNF	
other	TNFAIP6	TNF alpha induced protein 6	2,0	2,0	LPS	TNF	IL1B
other	BTG3	BTG family member 3	1,7	1,9		TNF	
other	FYB	FYN binding protein	1,6	1,9	LPS		
other	RALGDS	ral guanine nucleotide dissociation stimulator	2,0	1,9	LPS		

other	TFPI2	tissue factor pathway inhibitor 2		1,9	LPS	TNF	IL1B
other	GPR183	G protein-coupled receptor 183		1,8	LPS		
other	SOCS1	suppressor of cytokine signaling 1	1,6	1,8	LPS	TNF	IL1B
other	CYR61	cysteine rich angiogenic inducer 61		1,8	LPS	TNF	
other	RASA2	RAS p21 protein activator 2		1,8			IL1B
other	TRAF1	TNF receptor associated factor 1	1,6	1,8	LPS	TNF	IL1B
other	LCP1	lymphocyte cytosolic protein 1		1,8			IL1B
other	SERPINA3	serpin family A member 3	1,6	1,8	LPS	TNF	IL1B
other	TPM4	tropomyosin 4		1,7			
other	RGS2	regulator of G-protein signaling 2		1,7	LPS	TNF	
other	GADD45B	growth arrest and DNA damage inducible beta	1,6	1,6	LPS	TNF	IL1B
other	MYBPH	myosin binding protein H		1,6			
other	PVR	poliovirus receptor	1,6	1,6			
other	CD68	CD68 molecule		1,6			
other	GLIPR1	GLI pathogenesis related 1		1,5			
other	SERPINB8	serpin family B member 8		1,5		TNF	
other	APOLD1	apolipoprotein L domain containing 1	1,5	1,5			
other	ITGB6	integrin subunit beta 6	1,5	1,5		TNF	
other	HIST1H1D	histone cluster 1, H1d		-1,7			
other	KRT13	keratin 13		-1,8	LPS		
peptidase	ADAMTS4	ADAM metallopeptidase with thrombospondin type 1 motif 4	9,1	7,7		TNF	IL1B
peptidase	PLAU	plasminogen activator, urokinase	2,1	3,2	LPS	TNF	IL1B
peptidase	PLAT	plasminogen activator, tissue type	2,4	2,3	LPS	TNF	IL1B
peptidase	CASP7	caspase 7	1,8	2,1	LPS	TNF	
peptidase	CASP4	caspase 4		2,0	LPS	TNF	IL1B
peptidase	RAB7B	RAB7B, member RAS oncogene family	1,9	2,0			
peptidase	ADAMTS1	ADAM metallopeptidase with thrombospondin type 1 motif 1		1,5			IL1B
phosphatase	DUSP2	dual specificity phosphatase 2	3,3	3,6	LPS	TNF	

phosphatase	SOCS3	suppressor of cytokine signaling 3	3,3	2,9	LPS	TNF	IL1B
phosphatase	DUSP5	dual specificity phosphatase 5	2,6	2,7	LPS	TNF	IL1B
TR	IRF1	interferon regulatory factor 1	4,8	3,7	LPS	TNF	IL1B
TR	NFKBIA	NFKB inhibitor alpha	2,9	3,6	LPS	TNF	IL1B
TR	BCL3	B-cell CLL/lymphoma 3	3,8	3,2	LPS	TNF	IL1B
TR	ZFP36	ZFP36 ring finger protein	2,6	3,1	LPS	TNF	IL1B
TR	FOSL1	FOS like antigen 1	2,6	3,0		TNF	IL1B
TR	NFKBIZ	NFKB inhibitor zeta	1,7	3,0	LPS	TNF	IL1B
TR	NFKB1	nuclear factor kappa B subunit 1	2,4	2,7	LPS	TNF	IL1B
TR	SNAI1	snail family zinc finger 1	2,5	2,7		TNF	
TR	CEBPD	CCAAT/enhancer binding protein delta	2,4	2,6	LPS	TNF	IL1B
TR	FOSL2	FOS like antigen 2	2,1	2,4		TNF	
TR	MXD1	MAX dimerization protein 1	1,8	2,4	LPS		
TR	BHLHE40	basic helix-loop-helix family member e40	2,1	2,3		TNF	
TR	MYC	v-myc avian myelocytomatosis viral oncogene homolog	2,3	2,3	LPS	TNF	IL1B
TR	BTG2	BTG family member 2	1,7	2,2		TNF	IL1B
TR	NFIL3	nuclear factor, interleukin 3 regulated	2,0	2,2	LPS		IL1B
TR	NFKB2	nuclear factor kappa B subunit 2	1,9	2,2	LPS	TNF	IL1B
TR	PRDM1	PR domain 1	2,0	2,2	LPS	TNF	
TR	RCAN1	regulator of calcineurin 1	1,9	2,1	LPS	TNF	IL1B
TR	RELB	RELB proto-oncogene, NF-kB subunit	2,1	2,1	LPS	TNF	IL1B
TR	MSC	musculin		2,0	LPS	TNF	
TR	FOS	FBJ murine osteosarcoma viral oncogene homolog		1,9	LPS	TNF	IL1B
TR	NFKBIE	NFKB inhibitor epsilon	1,7	1,9	LPS	TNF	
TR	SBNO2	strawberry notch homolog 2 (Drosophila)		1,9			
TR	ETS2	ETS proto-oncogene 2, transcription factor	1,7	1,8	LPS		IL1B
TR	CSRNP1	cysteine and serine rich nuclear protein 1	1,7	1,8			IL1B
TR	NFKBID	NFKB inhibitor delta	1,5	1,8	LPS		
TR	SKIL	SKI-like proto-oncogene		1,7			

TR	KLF10	Kruppel-like factor 10	1,5	1,6	LPS	TNF	IL1B
TR	ZBTB16	zinc finger and BTB domain containing 16		-1,5			
TMR	PLAUR	plasminogen activator, urokinase receptor	4,8	5,9	LPS	TNF	
TMR	CD40	CD40 molecule	4,3	5,0	LPS	TNF	IL1B
TMR	ICAM1	intercellular adhesion molecule 1	5,6	4,8	LPS	TNF	IL1B
TMR	CD83	CD83 molecule	3,8	4,5	LPS	TNF	IL1B
TMR	SELP	selectin P	5,0	4,2	LPS	TNF	
TMR	CD69	CD69 molecule	3,1	3,8	LPS	TNF	IL1B
TMR	CSF3R	colony stimulating factor 3 receptor	3,8	3,8	LPS		
TMR	OLR1	oxidized low density lipoprotein receptor 1	2,4	3,3	LPS	TNF	IL1B
TMR	F3	coagulation factor III, tissue factor	2,6	2,8	LPS	TNF	IL1B
TMR	IGSF6	immunoglobulin superfamily member 6	2,1	2,6	LPS		
TMR	SELL	selectin L	2,4	2,6	LPS	TNF	
TMR	VCAM1	vascular cell adhesion molecule 1		2,6	LPS	TNF	IL1B
TMR	TLR2	toll like receptor 2	1,9	2,4	LPS	TNF	IL1B
TMR	IFNAR2	interferon alpha and beta receptor subunit 2	1,9	2,2		TNF	
TMR	IL1RAP	interleukin 1 receptor accessory protein		2,1	LPS		IL1B
TMR	SLAMF6	SLAM family member 6	1,8	2,1			
TMR	CD86	CD86 molecule		2,0	LPS	TNF	IL1B
TMR	CD80	CD80 molecule	1,6	1,8	LPS	TNF	IL1B
TMR	IL18R1	interleukin 18 receptor 1		1,7	LPS	TNF	IL1B
TMR	ITGB3	integrin subunit beta 3		1,6		TNF	IL1B
transporter	SLCO4A1	solute carrier organic anion transporter family member 4A1	3,0	3,0			
transporter	SLC2A3	solute carrier family 2 member 3	2,3	2,9			
transporter	STX11	syntaxin 11	2,7	2,8	LPS		
transporter	ABCC4	ATP binding cassette subfamily C member 4		2,1	LPS		
transporter	LBP	lipopolysaccharide binding protein	1,6	1,6	LPS	TNF	IL1B
transporter	SNX10	sorting nexin 10		1,6			

B) Genes, regulated only by *S. aureus*

Type(s)	Symbol	Gene Name	FC	Upstream regulator		
cytokine	DKK3	dickkopf WNT signaling pathway inhibitor 3	1,5			
enzyme	B3GALNT1	beta-1,3-N-acetylgalactosaminyltransferase 1	1,9			
enzyme	TGM2	transglutaminase 2	1,8	TGFB1		ESR1
enzyme	PLOD2	procollagen-lysine, 2-oxoglutarate 5-dioxygenase 2	1,7	TGFB1		ESR1
enzyme	PGM5	phosphoglucomutase 5	1,6			
enzyme	EXTL2	exostosin-like glycosyltransferase 2	1,6		SRF	
enzyme	FKBP7	FK506 binding protein 7	1,6			
enzyme	LOX	lysyl oxidase	1,6	TGFB1		
enzyme	RND3	Rho family GTPase 3	1,6		SRF	ESR1
enzyme	PDE5A	phosphodiesterase 5A	1,5			
enzyme	GPX3	glutathione peroxidase 3	1,5			
enzyme	FAH	fumarylacetoacetate hydrolase (fumarylacetoacetase)	-1,6			
enzyme	DIO2	deiodinase, iodothyronine, type II	-1,6			
enzyme	SCLY	selenocysteine lyase	-1,6			
GPCR	NPR3	natriuretic peptide receptor 3	1,7			ESR1
GPCR	GPR68	G protein-coupled receptor 68	-1,6			
GF	PTN	pleiotrophin	1,6			
GF	IGF1	insulin like growth factor 1	1,6	TGFB1	SRF	ESR1
kinase	RSPO3	R-spondin 3	2,0			
kinase	HSPB8	heat shock protein family B (small) member 8	1,6			
kinase	DCLK1	doublecortin like kinase 1	1,6			
kinase	MYLK	myosin light chain kinase	1,5	TGFB1	SRF	
kinase	CKB	creatine kinase, brain	1,5			ESR1
kinase	FGR	FGR proto-oncogene, Src family tyrosine kinase	-1,7			
microRNA	mir-143	microRNA 143	1,7			

other	ACTG2	actin, gamma 2, smooth muscle, enteric	2,0	TGFB1	SRF	
other	C1QTNF3	C1q and tumor necrosis factor related protein 3	1,9			
other	NCAM1	neural cell adhesion molecule 1	1,9	TGFB1		
other	SLITRK5	SLIT and NTRK like family member 5	1,8			
other	SYNPO2	synaptopodin 2	1,8			
other	MAP1B	microtubule associated protein 1B	1,8			ESR1
other	TPM2	tropomyosin 2 (beta)	1,8	TGFB1		
other	CNN1	calponin 1, basic, smooth muscle	1,7	TGFB1	SRF	ESR1
other	CFL2	cofilin 2 (muscle)	1,7			ESR1
other	ITGBL1	integrin subunit beta like 1	1,7	TGFB1		
other	PRND	prion protein 2 (dublet)	1,7			
other	TMEM45A	transmembrane protein 45A	1,7			
other	CTHRC1	collagen triple helix repeat containing 1	1,7			
other	DYNC2LI1	dynein, cytoplasmic 2, light intermediate chain 1	1,7			
other	CDC42EP3	CDC42 effector protein 3	1,6	TGFB1		ESR1
other	SGCB	sarcoglycan beta	1,6			
other	ISLR2	immunoglobulin superfamily containing leucine-rich repeat 2	1,6			
other	MXRA7	matrix-remodelling associated 7	1,6			
other	NKAIN3	Na+/K+ transporting ATPase interacting 3	1,6			
other	SPARCL1	SPARC like 1	1,6	TGFB1		
other	THBS4	thrombospondin 4	1,6			ESR1
other	ASPN	asporin	1,6	TGFB1		
other	COL3A1	collagen, type III, alpha 1	1,6	TGFB1		
other	BAG2	BCL2 associated athanogene 2	1, 6			
other	DDIT4L	DNA damage inducible transcript 4 like	1,6			
other	IFT81	intraflagellar transport 81	1,6			
other	RCN1	reticulocalbin 1	1,6			
other	SLIT2	slit guidance ligand 2	1,6			
other	SYNC	syncoilin, intermediate filament protein	1,6			

other	MYH11	myosin, heavy chain 11, smooth muscle	1,5	TGFB1	SRF	
other	SH3BGR	SH3 domain binding glutamate-rich protein	1,5			
other	SMOC2	SPARC related modular calcium binding 2	1,5	TGFB1		
other	COL8A1	collagen, type VIII, alpha 1	1,5	TGFB1		ESR1
other	FLNA	filamin A	1,5	TGFB1	SRF	ESR1
other	PI16	peptidase inhibitor 16	1,5			
other	PARP8	poly(ADP-ribose) polymerase family member 8	1,5			
other	PFN2	profilin 2	1,5	TGFB1		ESR1
other	PTGFRN	prostaglandin F2 receptor inhibitor	1,5			
other	PRUNE2	prune homolog 2 (Drosophila)	1,5			
other	ZNF521	zinc finger protein 521	1,5			
other	BAMBI	BMP and activin membrane-bound inhibitor	1,5	TGFB1		
other	MAP1LC3B	microtubule associated protein 1 light chain 3 beta	-1,7			
peptidase	CPE	carboxypeptidase E	1,9			ESR1
phosphatase	ALPL	alkaline phosphatase, liver/bone/kidney	-1,6			
TR	EMX2	empty spiracles homeobox 2	1,7			
TR	MEIS1	Meis homeobox 1	1,7		SRF	
TR	MEIS2	Meis homeobox 2	1,7			
TR	BHLHE41	basic helix-loop-helix family member e41	1,6			
TR	MSX2	msh homeobox 2	1,6			
TR	TBX5	T-box 5	1,6			
TR	PRRX1	paired related homeobox 1	1,5			
TR	SMAD9	SMAD family member 9	1,5			
TR	SREBF1	sterol regulatory element binding transcription factor 1	-1,5			
TMR	SEMA7A	semaphorin 7A (John Milton Hagen blood group)	-1,7	TGFB1		
transporter	SLC6A17	solute carrier family 6 (neutral amino acid transporter), member 17	1,6			
transporter	PLN	phospholamban	1,5			
transporter	TRPA1	transient receptor potential cation channel, subfamily A, member 1	1,5			

Supplementary Table 4. Factors involved in cytoskeleton signalling regulated exclusively during *S. aureus* infection. List of DEGs and their fold changes encoding cytoskeleton associated factors as identified by the Ingenuity software.

Symbol	Entrez Gene Name	Exp Fold Change
ACTG2	actin, gamma 2, smooth muscle, enteric	2.0
RSPO3	R-spondin 3	2.0
NCAM1	neural cell adhesion molecule 1	1.9
SLITRK5	SLIT and NTRK like family member 5	1.8
TGM2	transglutaminase 2	1.8
SYNPO2	synaptopodin 2	1.8
MAP1B	microtubule associated protein 1B	1.8
TPM2	tropomyosin 2 (beta)	1.8
CFL2	cofilin 2	1.7
CTHRC1	collagen triple helix repeat containing 1	1.7
DYNC2LI1	dynein cytoplasmic 2 light intermediate chain 1	1.7
SGCB	sarcoglycan beta	1.6
CDC42EP3	CDC42 effector protein 3 (*alias* BORG2)	1.6
IGF1	insulin like growth factor 1	1.6
ISLR2	immunoglobulin superfamily containing leucine-rich repeat 2	1.6
LOX	lysyl oxidase	1.6
THBS4	thrombospondin 4	1.6
COL3A1	collagen type III alpha 1	1.6
RND3	Rho family GTPase 3	1.6
DCLK1	doublecortin like kinase 1	1.6
IFT81	intraflagellar transport 81	1.6
SLIT2	slit guidance ligand 2	1.6
MYH11	myosin, heavy chain 11, smooth muscle	1.5
MYLK	myosin light chain kinase	1.5
DKK3	dickkopf WNT signaling pathway inhibitor 3	1.5
FLNA	filamin A	1.5
PFN2	profilin 2	1.5
PRUNE2	prune homolog 2 (Drosophila)	1.5
PTGFRN	prostaglandin F2 receptor inhibitor	1.5
CKB	creatine kinase B	1.5
SREBF1	sterol regulatory element binding transcription factor 1	-1.5

MAP1LC3B	microtubule associated protein 1 light chain 3 beta (LOC783185)	-1.7
FGR	FGR proto-oncogene, Src family tyrosine kinase	-1.7
SEMA7A	semaphorin 7A (John Milton Hagen blood group)	-1.7

Supplementary Video. *S. aureus* invasion modulates the actin cytoskeleton in pbMEC. Video displaying in consecutive order optical sections (from bottom to top; section depth, 0.53 µM) of pbMEC harbouring invaded GFP-tagged *S. aureus* pathogens (green) and showing phalloidin stained actin fibres (red). The central cell harbours >10 *S. aureus* pathogens and displays thinner and longer actin fibres than those cells immediately adjacent to the left or right (located at 9 or 3 o'clock). These cells do not harbour *S. aureus* pathogens and they display thick ventral bundles of actin fibres being terminated by adhesion foci. Note that the cells located at around 6 o'clock also harbour single or few green fluorescing *S. aureus* pathogens. (Scale bar, 10 µm).

Supplementary video.avi
